# Ontogeny and Molecular Phylogeny of *Apoamphisiella vernalis* Reveal Unclear Separation between Genera *Apoamphisiella* and *Paraurostyla* (Protozoa, Ciliophora, Hypotricha)

**DOI:** 10.1371/journal.pone.0155825

**Published:** 2016-05-19

**Authors:** Larissa Araguaia Monteiro de Castro, Gabriela Cristina Küppers, Noemi Mendes Fernandes, Martin Schlegel, Thiago da Silva Paiva

**Affiliations:** 1 Laboratório de Biologia Molecular “Francisco Mauro Salzano”, Instituto de Ciências Biológicas, Universidade Federal do Pará, Belém, PA, Brazil; 2 División Invertebrados, Museo Argentino de Ciencias Naturales “Bernardino Rivadavia”, Buenos Aires, Argentina; 3 Laboratório de Biologia Evolutiva Teórica e Aplicada, Departamento de Genética, Universidade Federal do Rio de Janeiro, Rio de Janeiro, RJ, Brazil; 4 Molecular Evolution and Animal Systematics, Institute of Biology, University of Leipzig, Leipzig, Germany; 5 Laboratory of Evolutionary Protistology, Instituto de Biociências, Universidade de São Paulo, São Paulo, SP, Brazil; Laboratoire de Biologie du Développement de Villefranche-sur-Mer, FRANCE

## Abstract

Morphology and divisional morphogenesis of the hypotrich ciliate *Apoamphisiella vernalis* are investigated based on two populations from Brazil. Typical specimens of *A*. *vernalis* replicates its ventral ciliature from six fronto-ventral-transverse (FVT) anlagen independently formed for proter and opisthe, plus one or more short anlagen located between IV and V, which form surplus transverse cirri. Dorsal morphogenesis occurs as in typical oxytrichid dorsomarginalians, viz., with formation of three anlagen and fragmentation of the rightmost one. Dorsomarginal kineties are formed near anterior end of right marginal cirral row anlagen. Various anomalous specimens exhibiting more than two long ventral rows were found, which are explained by increasing the number of FVT anlagen and/or the number of cirri produced by anlagen. Comparative ontogeny and phylogenetic analyses based on the 18S rDNA reveal that *Apoamphisiella vernalis* is closely affine to North American and European strains of the *Paraurostyla weissei* complex. Their reduced genetic distances and conspicuous morphological variability show that both genera can overlap, which implies the necessity of re-evaluating the contextual relevance of some morphological characters commonly used for genus-level separation within hypotrich taxa.

## Introduction

The Hypotricha Stein, 1859 (= Stichotrichia Small & Lynn, 1985) is a relatively ubiquitous group of ciliates characterized by having, *inter alia*, a flat body with well differentiated ventral and dorsal regions, ventral ciliature organized in clusters and/or rows of locomotory cirri, dorsal ciliature with sensory bristles, at least one left and one right marginal cirral rows, a polyhymenophore adoral zone of membranelles and two undulating membranes (paroral and endoral) [1–5]. They occur in soils, freshwater and marine sediments, functioning usually as *K*-selected, omnivorous consumers which are important indicators for assessment of soil and water quality [6–9].

The systematics of Hypotricha is still one of the most unresolved issues within the phylum Ciliophora Doflein, 1901, which is sometimes attributed to innumerable convergences in ciliature patterns produced by diverse ontogenetic processes [5, 8]. Hence, the study of these ontogenetic processes is considered of major importance to explain how such ciliature patterns develop and organize, facilitating the detection of homologies among structures [10–23]. Variations in the number, position and number of structures produced by different anlagen (= primordia), like fronto-ventral-transverse (FVT), marginal and dorsal anlagen, are responsible for archetypical ciliature elements, such as the amphisiellid rows, the midventral complex, the oxytrichid “18-FVT” cirral pattern, and the many dorsal bristle arrangements that can be observed among different groups. Those are hitherto used not only for determination of hypotrich genera and species, but also their miscellaneous suprageneric ranks [1–4, 17, 19, 24–30]. Within the molecular context, the low resolution of hypotrich internal affinities is associated to low phylogenetic signal of molecular markers (in special the 18S rDNA) at suprageneric levels, which except for the urostyloids, tend to result in poorly resolved and/or supported topologies, e.g. [31–35]. According to Jung *et al*. [36], other possible explanations for the “difficult” systematics of hypotrichs may relate to misidentified sequences deposited in the NCBI/GenBank, inadequacy of methods, insufficient taxa sample and incorrect morphological concepts of taxonomic groups.

Intending to fill in a gap of the jigsaw puzzle of hypotrichs’ natural history, we investigate the morphology and ontogeny during divisional morphogenesis of *Apoamphisiella* Foissner, 1997, based on two Brazilian strains of *A*. *vernalis* (Stokes 1887) Berger 2006, and hypothesize its phylogenetic affinities from analyses of the 18S-rDNA. A remarkable interphase variability is found in *A*. *vernalis*, which is explained from ontogeny, and that overlaps with the diagnostic features of *Paraurostyla* Borror, 1972, a genus already supposed to be closely related to *Apoamphisiella* [1].

## Materials and Methods

### Environmental sampling and morphological methods

The specimens from Pará (PA) were found in water samples with muddy sediments, collected at the margins of the Tucunduba river, located in Guamá, a district in the city of Belém, capital of the state of Pará, Brazil (geographic coordinates: -1.473919, -48.455029), in December of 2013. Samples were collected with sterilized glass flasks and brought to the laboratory on the same sampling date. Initially, cultures were made in glass Petri dishes from aliquots of raw samples with addition of crushed rice grains to promote bacterial growth, which serve as primary food source for the ciliates [8, 28]. Subsequent cultures were established using mineral water instead of water from the sampling site. For morphological characterization and study of morphogenesis in the population from PA, specimens from non-clonal cultures were used.

Ciliates were first studied *in vivo* under stereomicroscope, and then using brightfield and differential interference contrast (DIC) at 100×, 200× (coverless slides), 400× (with a cover slip) and 1,000× (oil immersion). Protargol impregnation [37] was used to reveal the nuclear apparatus, infraciliature and to study morphogenesis during binary fission. Scanning electron microscopy (SEM) preparations were made following da Silva-Neto *et al*. [38] to obtain additional information on the external morphology of the ciliates.

The specimens from Minas Gerais (MG) used in the present study were found in water samples with sediments from a shallow pond of the Atlantic Forest in the vicinity of the city of Caxambú, in the state of Minas Gerais, Brazil, in October of 2006 (approximate geographic coordinates: -21.993451, -44.954921). That is the same sampling site mentioned by Paiva *et al*. [39], who studied the morphology of a population of *A*. *vernalis* previously obtained in 2002. Collection, cultivation (in raw, limnetic cultures), live observations, protargol impregnation [40], isolation and fixation of specimens for DNA extraction were made by T. da S. Paiva in 2006, following the methodologies mentioned in Paiva & da Silva-Neto [28]. Since the typical specimens of this population are basically identical to those from 2002, the reader is referred to Paiva *et al*. [39] for the interphase morphology of *A*. *vernalis* from MG. Some deviations, not reported in that study because they were not observed in the population from 2002, are mentioned and discussed in the present paper.

No specific permissions were required to collect the material, because it was obtained from public locations. No known endangered or protected species were involved in the present study.

Measurements of protargol-impregnated specimens were carried out with an ocular micrometer (Table 1). Drawings of interphase and dividing specimens of *A*. *vernalis* were performed at 1,000× magnification with aid of a drawing device. Parental structures are depicted by contour; anlagen and juvenile structures are shaded black. Length of cilia were measured from scanning electron images, cytoplasmic inclusions were measured *in vivo*, and measurements of other structures are from protargol-impregnated specimens. Drawings and micrographs of *A*. *vernalis* are from specimens of the population from PA, unless otherwise mentioned.

**Table 1:** Morphometric data on *Apoamphisiella vernalis* from Belém-PA, Brazil.

Character	Mean	M	Min	Max	SD	CV(%)	n
Body, length	218.5	212.5	150.0	305.0	41.9	19.2	20
Body, width	81.8	82.5	43.0	100.0	15.0	18.2	20
AZM, length	92.5	92.5	70.0	120.0	17.8	19.2	20
Macronuclear nodules, number	2.0	2.0	2	3	0.2	11.0	20
Anterior macronuclear nodule, length	37.7	38.0	26.0	50.0	7.5	20.0	20
Anterior macronuclear nodule, width	18.7	17.7	14.0	26.0	3.3	17.7	20
Posterior macronuclear nodule, length	36.8	37.5	26.0	55.0	8.1	22.1	20
Posterior macronuclear nodule, width	19.4	18.5	14.0	28.0	3.8	19.8	20
Micronuclei, number	3.1	3.0	2	6	1.0	32.1	21
Micronucleus, length	7.6	7.7	5.5	10.0	1.2	16.2	20
Micronucleus, width	6.6	6.7	5.0	8.0	0.9	14.2	20
Membranelles, number	56.3	56.0	50	62	2.9	5.1	20
Frontal cirri, number	3.0	3.0	3	3	0.0	0.0	20
Buccal cirri, number	1.0	1.0	1	1	0.0	0.0	20
Frontoventral cirri, number	2.0	2.0	2	2	0.0	0.0	20
Postperistomal cirri, number	1.1	1.0	1	2	0.3	28	20
Right ventral row, number of cirri	30.6	30.0	28	35	2.1	7.0	20
Left ventral row, number of cirri	21.8	22.0	19	24	1.4	6.5	20
Transverse cirri, number	6.2	6.0	5	8	0.6	10.0	21
Right marginal row, number of cirri	38.8	37.5	33	48	4.5	11.7	20
Left marginal row, number of cirri	36.6	36.0	32	43	3.4	9.2	20
Dorsal kineties, number	4.0	4.0	4	4	0.0	0.0	8
Dorsomarginal kineties, number	2.6	3.0	2	3	0.5	19.7	8
Total caudal cirri, number	8.8	9.0	7	11	0.2	11.7	20
Caudal cirri at end of DK1, number	2.5	2.0	2	4	0.1	27.5	20
Caudal cirri at end of DK2, number	2.1	2.0	2	3	0.1	17.0	20
Caudal cirri at end of DK4, number	4.2	4.0	3	7	0.2	21.3	20

Measurements are in μm. AZM = adoral zone of membranelles; CV = coefficient of variation; DK(n) dorsal kinety; M = median; Mean = arithmetic mean; n = sample size; SD = standard deviation.

The stylized diagrams showing homologies among ventral cirri in *Cyrtohymena*, *P*. *weissei* and *Apoamphisiella* were made with Adobe Photoshop CS5 (Adobe Systems Inc., USA), and were based on data from Singh & Kamra [41], Jerka-Dziadosz & Frankel [12], Wirnsberger *et al*. [42] and Foissner *et al*. [9]. Hypotheses of homology among ventral cirral patterns were grounded on anlagen behavior (i.e., type of cirri formed, migratory pattern and position of resulting cirri) and topology in comparable semaphoronts.

Frontoventral-transvese (FVT) anlagen numbering is based on the system established by Wallengren [43]. Scale bars were used when necessary (see Foissner & Xu [44] p.55, for a brief, but very proficuous discussion on this matter). Terminology follows Berger [1] and Paiva *et al*. [39]. The term “serial homology” is borrowed from the old definition of Owen [45–47], as the presence of corresponding morphological structures in different parts of the body. It is herein applied to the occurrence of serially repeated, corresponding cirral anlagen that exhibit similar migratory behaviour and produce similar cirral structures (even though some modifications/specializations might occur within the series).

### Extraction of DNA and gene amplification

Approximately 30 specimens were picked from clonal cultures (i.e. culture started from a single specimen) of the population from PA, washed in sterilized mineral water, and fixed with 70% ethanol. Specimens from MG were isolated and fixed in 2006, and stored at -20°C. The phenol-chloroform method following Sambrook *et al*. [48] was used for DNA extraction, and amplification of 18S rDNA was performed using universal eukaryotic primers Euk A (5′-ACCTGGTTGATCCTGCCAGT-3′) and Euk B (5′ TGATCCTTCTGCAGGTTCACCTAC-3') [49].

The obtained 25 μl PCR mix contained: 14.8 μl of HPLC H_2_O, 2.5 μl of 10x Dream Taq Green Buffer^®^, 0.2 μl of DreamTaq^®^ DNA Polymerase (both from Thermo Scientific, Vienna, Austria), 2.5 μl of 10 μM dNTPs, 1.0 μl of each primer (10 μM stock concentration), and 3.0 μl of the extracted DNA. The PCR amplifications were performed in a Nexus Mastercycler (Eppendorf North America, New York, USA). Amplification cycles were as follows: 5 min at 94°C followed by 30 cycles of 94°C for 1 min, 60°C for 1 min 15 s, 72°C for 1 min 30 s and a final extension at 72°C for 10 min. The PCR products were purified using the NucleoSpin Gel and PCR Clean-up Kit (Macherey-Nagel, Düren, Germany) according to the manufacturer’s protocol.

Purified PCR products of expected size (~1,800 bp) were ligated into pGEM T-easy Vector (Promega, Mannheim, Germany) and cloned into competent *E*. *coli* JM109 cells. After incubation time, white colonies were selected for PCR with M13 forward (5'-TGTAAAACGACGGCCAGT-3') and M13 reverse (5'-CAGGAAACAGCTATGAC-3') primers. The cycles for this PCR reaction were: 3 min at 94°C followed by 35 cycles of 94°C for 30 s, 55°C for 30 s, 72°C for 1 min 30 s and a final extension at 72°C for 5 min. After amplification, the PCR product from M13 primers were visualized in agarose gel, and among those with expected size (~1,800 bp), five clones of each population sample were selected for purification and sequencing at GATC Biotech (Konstanz, Germany). The resulting data were assembled in contigs, analyzed and edited in the computer program Geneious 7.1.7 (http://www.geneious.com/).

### Taxa sample and 18S rDNA matrix alignment

One sequence of the population from PA and two from MG were obtained from cloning. They were added to a nucleotide matrix with other 18S rDNA sequences downloaded from the NCBI/GenBank, summing 64 terminals. The ingroup terminals were sampled within the non-urostyloid Hypotricha [3, 4], emphasizing those found to be immediately similar to *A*. *vernalis* after an initial BLAST [50] search, viz. representatives of the Dorsomarginalia Berger, 2006. Special attention was given to the *Cyrtohymena*-*Paraurostyla* group, discussed in Foissner *et al*. [51], because *Paraurostyla* is supposed to be closely related to *Apoamphisiella* [1]. Thus, all 18S rDNA sequences identified as *Paraurostyla* available in the NCBI/GenBank on January of 2016 were included in the matrix. Those were the members of the *P*. *weissei* complex, AJ310485 (Austria), AF164127 and AY294648 (Princeton, New Jersey, USA), and AF508767 (Boulder, Colorado, USA); and *P*. *viridis* AF508766 (Sarasota, Florida, USA). Sequences of other non-urostyloid hypotrichs of unresolved affinities according to previous studies, e.g. [35, 36], such as gonostomatids, kahliellids, some oxytrichids and spirofilids, were included in the ingroup to increase taxon sample outside the typical Dorsomarginalia. Six sequences of urostyloids were added as outgroup. The sequences were aligned using the MUSCLE algorithm (with its default parameters) implemented in the computer program MEGA 6 [52], with which they were inspected and trimmed at both ends. Ambiguously aligned sites were manually selected and re-aligned first by MUSCLE (using the preset configuration for refining alignment), and then by eye. A pairwise distance matrix (Table 2) was calculated in MEGA 6.

**Table 2:** Uncorrected *P*-distances among the terminals in the cluster of the “non-stylonychine dorsomarginalians” in which *A*. *vernalis* was placed.

		1	2	3	4	5	6	7	8
**1**	*Apoamphisiella vernalis* MG1								
**2**	*Apoamphisiella vernalis* MG2	0.002							
**3**	*Apoamphisiella vernalis* PA	0.004	0.002						
**4**	*Notohymena apoaustralis* KC430934	0.004	0.002	0.004					
**5**	*Paraurostyla viridis* AF508766	0.038	0.037	0.038	0.035				
**6**	*Paraurostyla weissei* AF164127	0.003	0.001	0.003	0.004	0.037			
**7**	*Paraurostyla weissei* AF508767	0.003	0.001	0.003	0.004	0.037	0.000		
**8**	*Paraurostyla weissei* AJ310485	0.005	0.003	0.005	0.003	0.035	0.004	0.004	
**9**	*Paraurostyla weissei* AY294648	0.003	0.001	0.003	0.004	0.037	0.000	0.000	0.004

*Paraurostyla viridis* was included for comparison. GenBank accession codes given right of species names. MG1 and MG2 = clones of the population from Minas Gerais, collected in 2006; PA = population from Pará.

### Phylogenetic analyses

Phylogenetic analyses included Bayesian inference (BI) and maximum-likelihood (ML) methods. The BI employed the GTR + I + Γ model of nucleotide substitution, selected according to Akaike information criterion [53, 54] used in MrModeltest 2.3 [55]. The inferences was performed in MrBayes 3.2.2 [56], implemented in the CIPRES Science Gateway [57], and was based on two independent Markov Chain Monte Carlo (MCMC) simulations run with four chains of 5,000,000 generations. Tree samples were taken each 5,000 generations, and their first 25% were discarded as burn-in. For ML, the data were analyzed using PhyML3.0 [58] using the GTR + I + Γ model, with empirical values for Γ shape and proportion of variable sites. The analysis started from an initial BioNJ dendrogram, of which likelihood was improved via subtree pruning and regrafting (SPR) branch-swapping moves to achieve the ML tree.

Support of branching patterns was estimated from posterior probabilities in BI (calculated from 50% majority rule consensus of trees retained after burn-in), and 1,000 bootstrap pseudoreplicates in ML [59]. Tree rooting was performed *a posteriori*, according to the outgroup position [60].

Among the selected ingroup sequences, the identity of *P*. *viridis* is questionable [2, 32] due to its phylogenetic position in previous studies, remarkably distant from the *P*. *weissei* complex, and next to *Oxytricha granulifera* [32, 61–63]. Thus, statistical procedures, viz. approximately unbiased (AU), Shimodaira-Hasegawa (SH) and weighted Shimodaira-Hasegawa (WSH), implemented in the computer program CONSEL [64], were performed to test whether trees with constrained topologies to enforce the monophyly of *Paraurostyla* (Table 2) differed significantly from the ML tree.

The taxonomy of *Apoamphisiella vernalis* is presented in the results below according to the system of Berger [2]. However, the systematics of Hypotricha is still a rather confused subject [2, 4, 5, 8]. The results on 18S rDNA phylogeny of hypotrichs present in the (vast) literature published in the last years, e.g. [31, 32, 35, 36, 51, 65, 66], repeatedly show that fitting the morphological/traditional Oxytrichidae and Urostyloidea (which were the first groups to appear in the early molecular papers) in the context of such trees is not possible, because the former spreads across most non-urostyloid nodes, and the latter interpolates with amphisiellids, gonostomatids, kahliellids, spirofilids and trachelostylids. Consequently, monophyly of virtually every traditional hypotrich taxa of ordinal or familial level is disrupted. Thus, we preferred not to address to the clusters hypothesized in the present study by names of non-monophyletic taxa, for which the circumscription changes according to *varia sensibus*. Instead, in the presented phylogenetic tree, we restricted labeling of relevant groups to a minimum necessary for communication, using vernacular names that can be easily abandoned if proved inappropriate, or legitimated with formal erection of new taxa in the future.

## Results

Spirotrichea Bütschli, 1889

Hypotricha Stein, 1859 (= Stichotrichia Small & Lynn, 1985)

*Incertae sedis* in Dorsomarginalia Berger, 2006

*Apoamphisiella* Foissner, 1997

*Apoamphisiella vernalis* (Stokes, 1887) Berger, 2006 [original combination: *Holosticha vernalis* Stokes, 1887]

### Morphology of *A*. *vernalis* from the Tucunduba river, Belém, PA.

**Fig 1:**
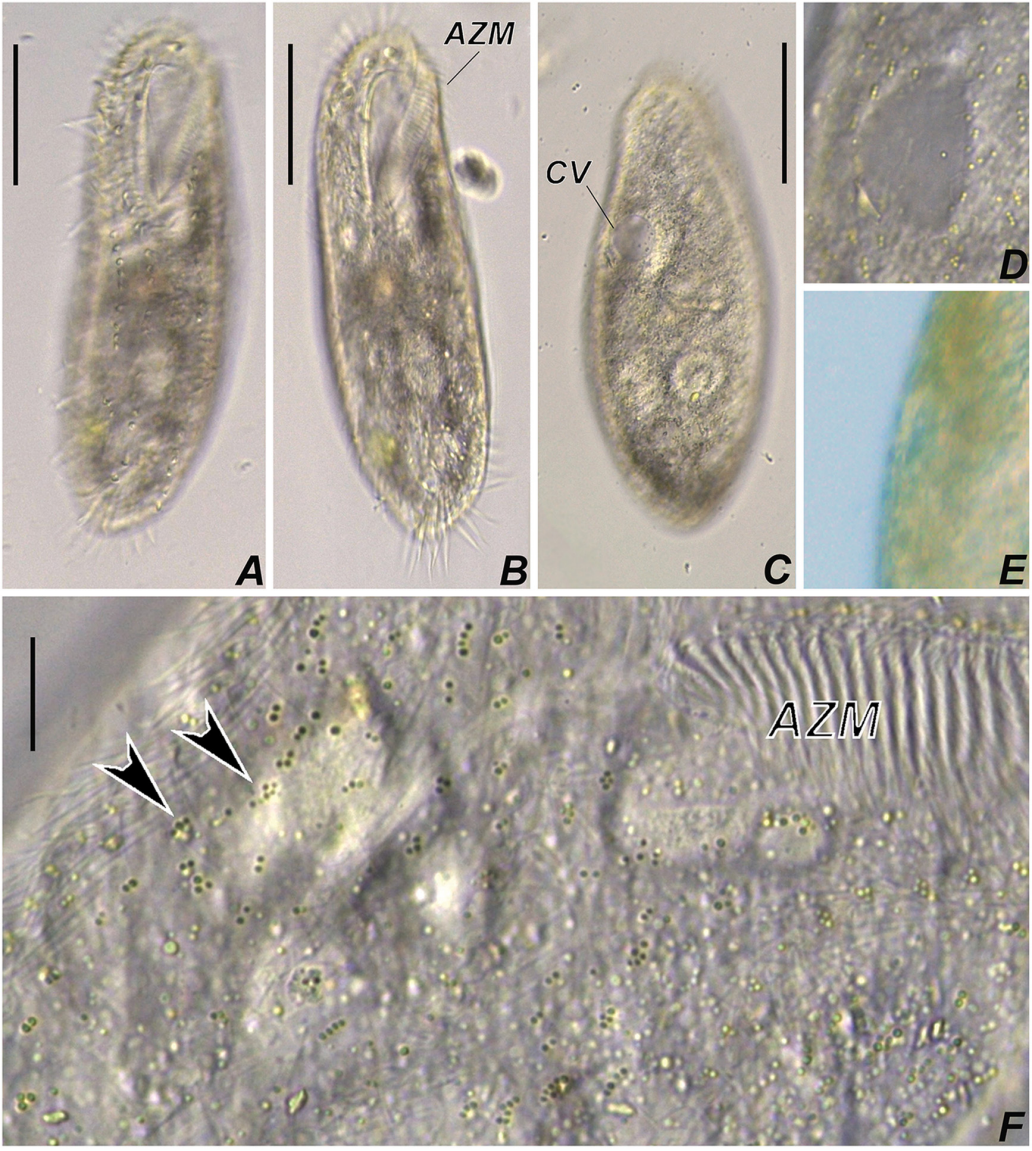
Photomicrograhps of *Apoamphisiella vernalis* from life under bright field. (A, B) Ventral side. (C) Dorsal side of slightly contracted specimen. (D, E) Detail of contractile vacuole; systole in (E). (F) Ventral side, to show cortical granules (arrowheads). AZM, adoral zone of membranelles; CV, contractile vacuole. Scale bars: A, B, C. 50 μm; F. 10 μm.

**Fig 2:**
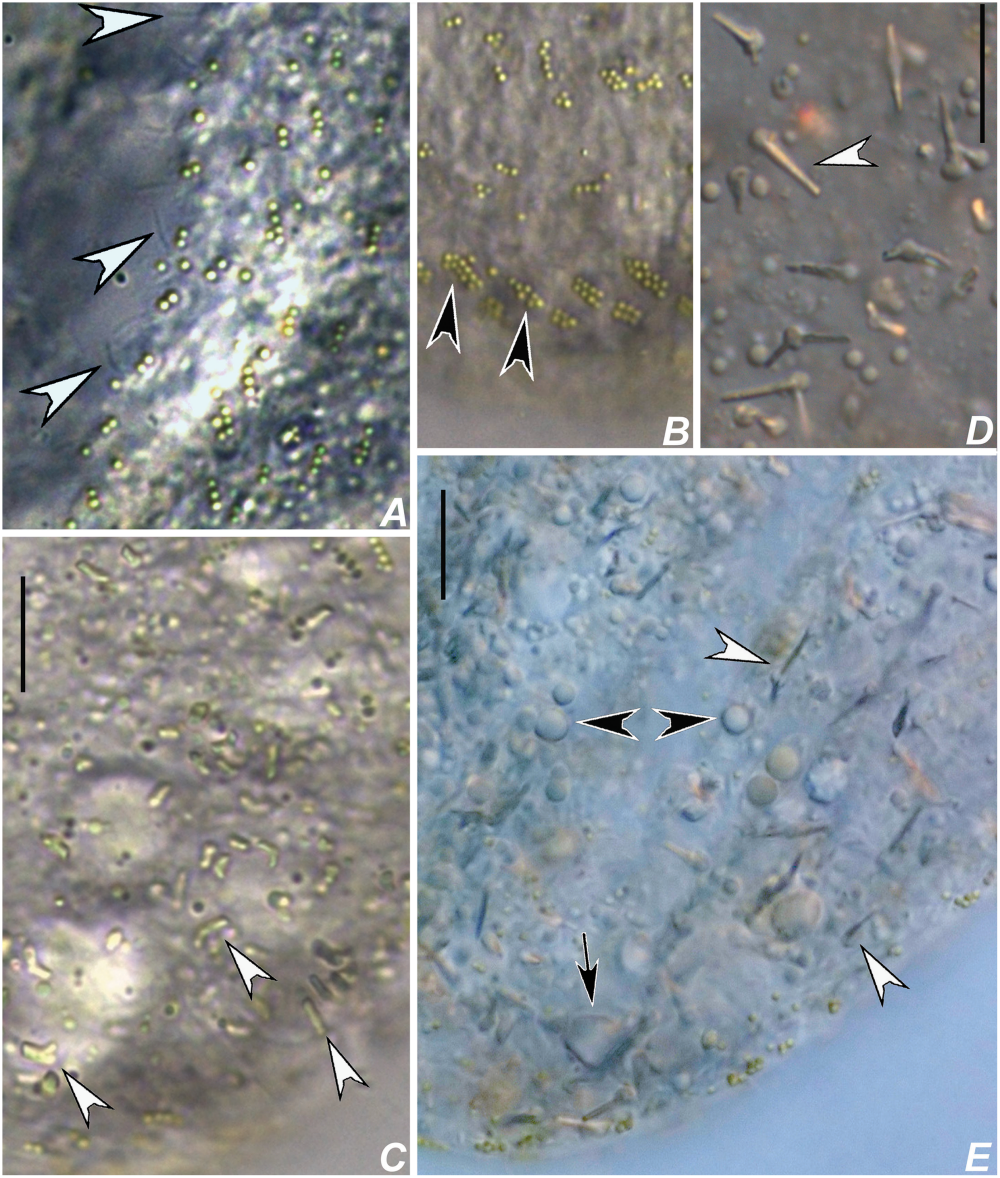
Photomicrograhps of *Apoamphisiella vernalis*, from life with DIC (A, D, E) and bright field (B, C). (A) Cortical granules on dorsal side of body. Arrowheads show dorsal bristles. (B) Clusters of cortical granules near caudal cirri (arrowheads). (C) Aculeate crystals of specimen from a mineral water culture, after cell rupture. (D, E) Cytoplasmic crystals (arrowheads) inside specimens from raw samples of Tucunduba river (D) and from a ruptured cell from a culture made with mineral water (E); black arrowheads show lipid droplets, white arrowheads mark aculeate crystals, and arrow shows a polygonal crystal. Scale bars: 10 μm.

**Fig 3:**
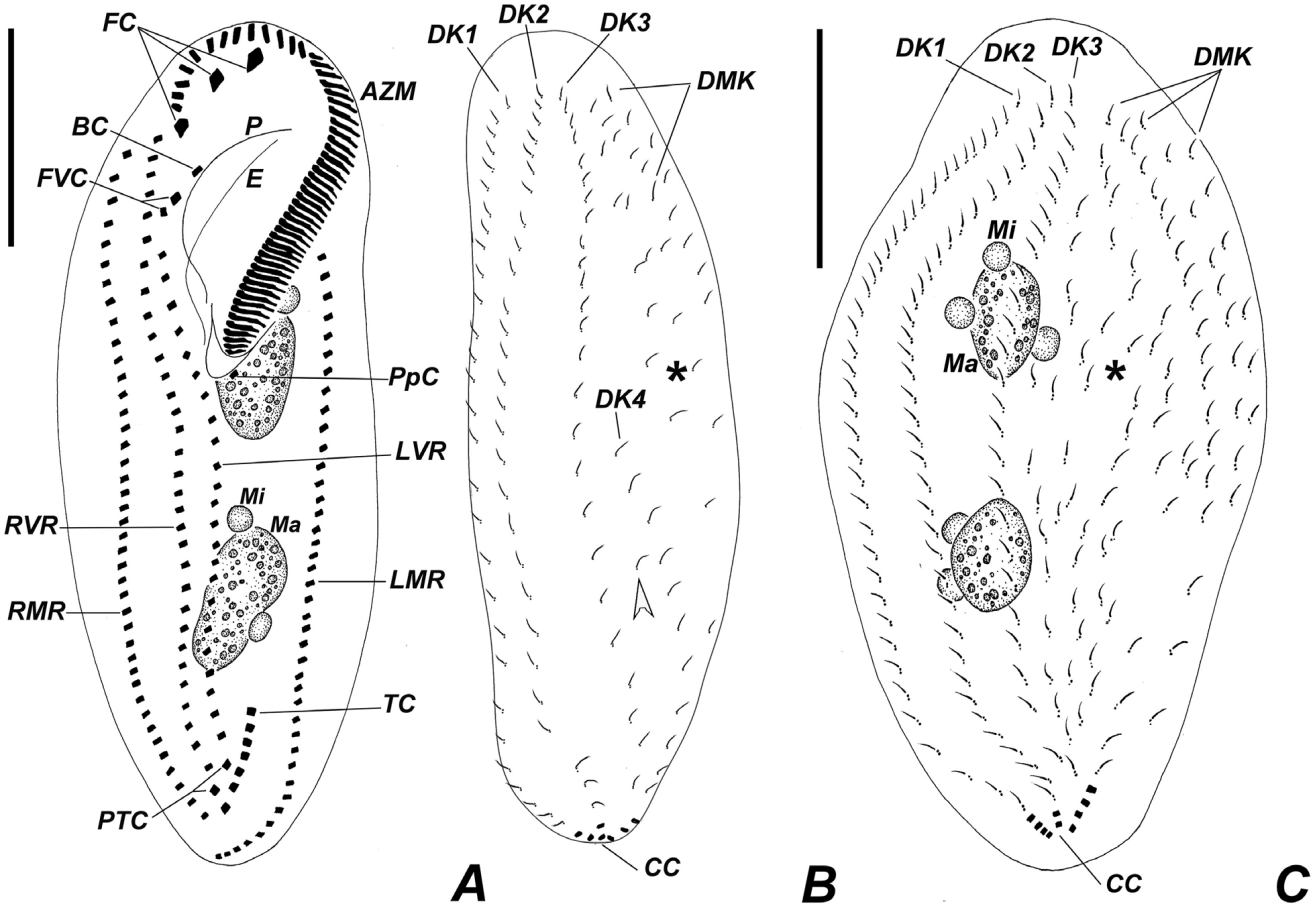
Diagrammatic representations of protargol-impregnated specimens of *Apoamphisiella vernalis*. (A) Ventral side. (B) Dorsal side of typical specimen. Asterisk marks field of scattered dikinetids; white arrowhead shows a scattered dikinetid located between dorsal kineties 3 and 4. (C) Dorsal side of specimen with supernumerary scattered dikinetids (asterisk), possibly an outlier. AZM = adoral zone of membranelles; BC = buccal cirrus; CC = caudal cirri; DK(n) = dorsal kineties; DMK = dorsomarginal kineties; E = endoral; FC = frontal cirri; FVC = frontoventral cirri; LMR = left marginal cirral row; LVR = left ventral cirral row; Ma = macronuclear nodule; Mi = micronucleus; P = paroral; PpC = postperistomal cirrus; PTC = pretransverse cirri; RMR = right marginal cirral row; RVR = right ventral cirral row; TC = transverse cirri. Scale bars: 50 μm.

Size *in vivo* about 180–210 μm × 50–65 μm; length to width ratio *in vivo* 3.2:1 and after protargol impregnation 2.7:1 (Table 1); outline elongate elliptical, anteriorly and posteriorly rounded, sometimes slightly narrowed anteriorly; dorsoventrally flattened about 2:1. Body flexible and only slightly contractile. Contractile vacuole on left body margin at level of buccal vertex, spherical when diastolic but becomes elliptical during systole; collecting canals either absent or extremely inconspicuous. Cortical granules green-colored, ~ 0.3–0.5 μm in diameter, located along and between cirral rows ventrally, but more conspicuously along dorsal kineties. Granules usually arranged in groups of three or four, but in larger clusters near caudal cirri. Cytoplasm transparent, with several refractive inclusions that renders a dark coloration at low magnification (40×, 100×), such as lipid droplets, transparent polygonal crystals, yellowish rod and “L”-shaped ~ 1–6 μm long crystals. The latter ones tend to have aculeate edges and appear to be more numerous and conspicuous in specimens cultivated in mineral water than in the original sample (Figs 1A–1F and 2A–2E). Normally with two ellipsoidal macronuclear nodules left of midbody and three globular micronuclei (Fig 3A and 3C).

**Fig 4:**
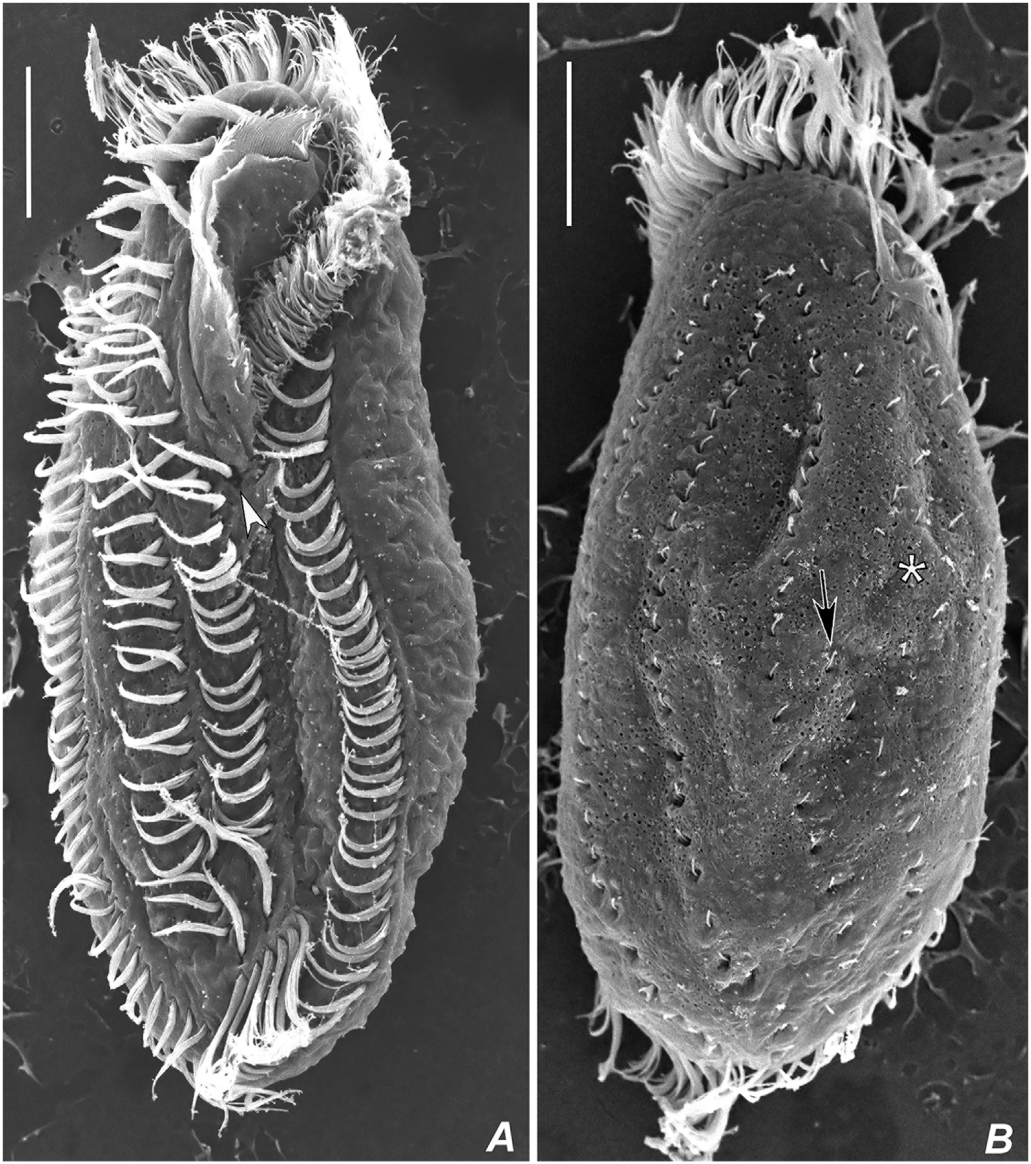
Scanning electron micrographs of *Apoamphisiella vernalis*. (A) Ventral side. Arrowhead points to postperistomal cirrus. (B) Dorsal side. Asterisk marks scattered dikinetids; arrow shows anterior end of dorsal kinety 4. Scale bars: 20 μm.

**Fig 5:**
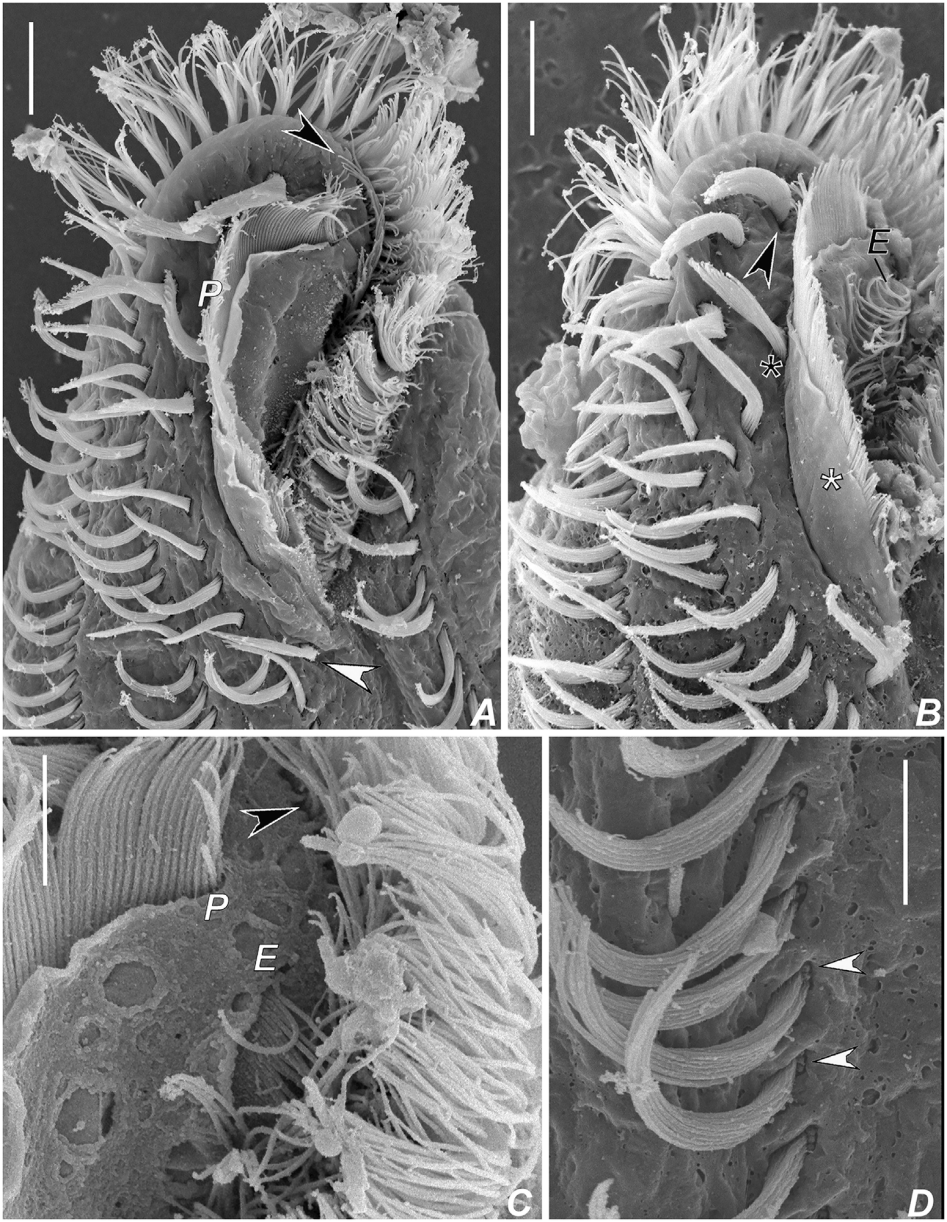
Scanning electron micrographs of *Apoamphisiella vernalis*. (A–C) Anterior region of ventral side. (A) Black arrowhead shows lateral membranellar cilia; white arrowhead shows postperistomal cirrus. (B) Black arrowhead shows leftmost frontal cirrus; black asterisk marks buccal cirrus; white asterisk marks oral lip. (C) Detail of distal ends of endoral and paroral. Arrowhead shows lateral membranellar cilia. (D) Detail of left marginal cirri to show barren basal bodies (arrowheads). E = endoral; P = paroral. Scale bars: A, B. 10 μm; C, D. 5 μm.

**Fig 6:**
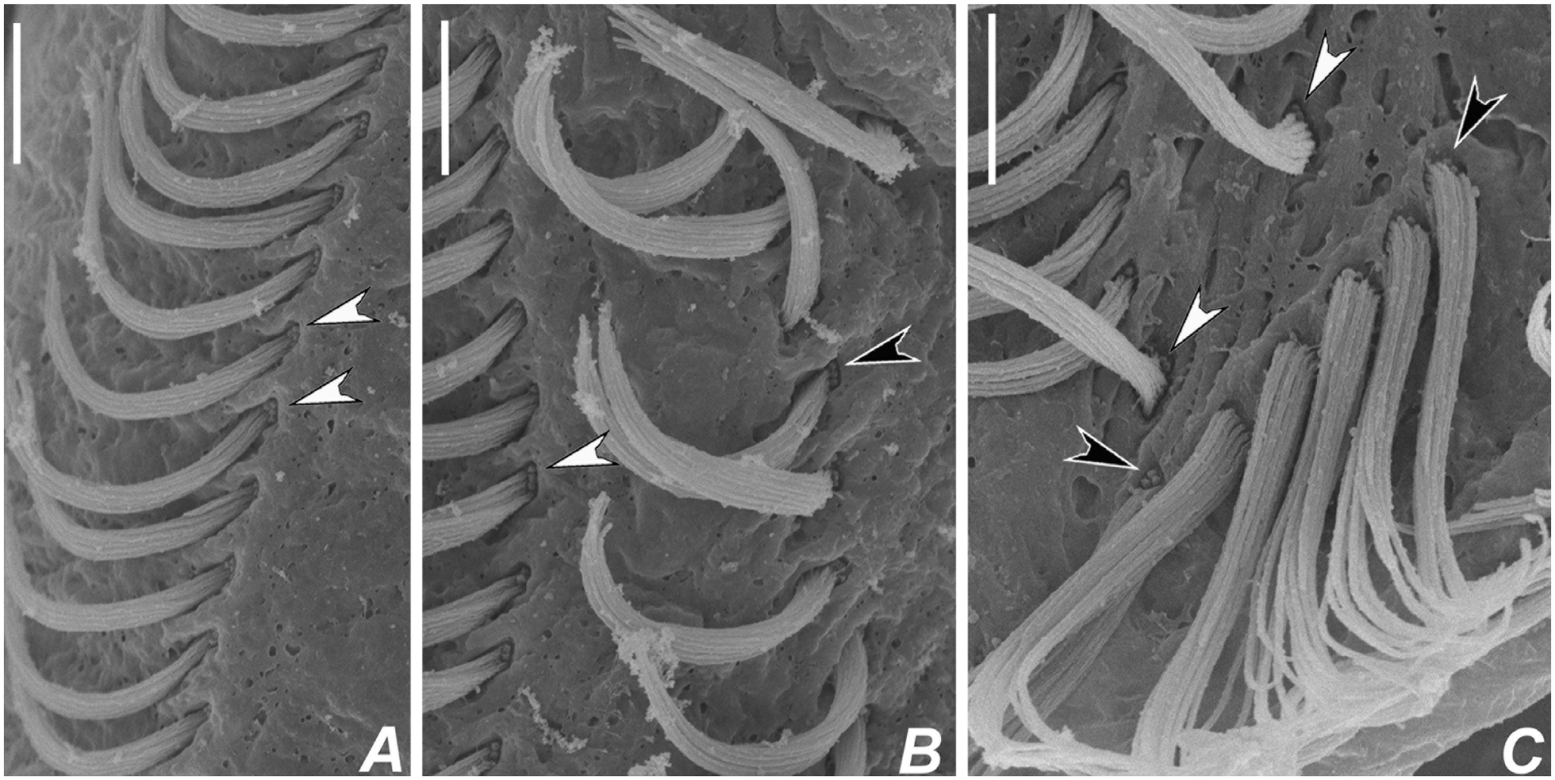
Scanning electron micrographs of *Apoamphisiella vernalis* on ventral side. (A) Right marginal cirral row. Arrowheads show barren basal bodies. (B) Ventral rows. White arrowhead points to barren basal bodies in the left row; black arrowhead in the right row. (C) Barren basal bodies in pretransverse (white arrowhead) and transverse cirri (black arrowhead). Scale bars: 5 μm.

**Fig 7:**
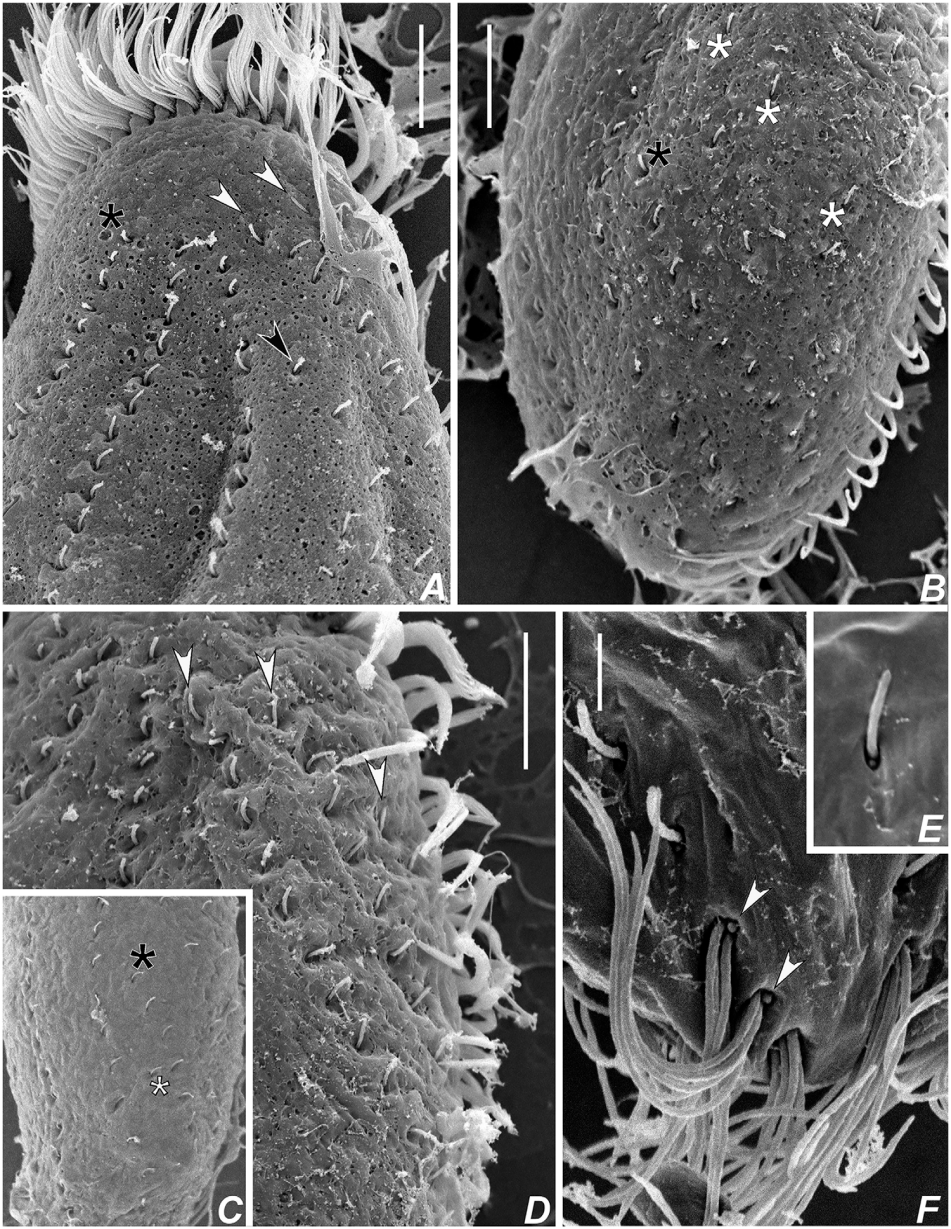
Scanning electron micrographs of *Apoamphisiella vernalis* on dorsal side. (A) Anterior region. Black arrowhead points to dorsomarginal kinety with relatively wider-spaced kinetosomes, thus possibly a retained parental structure. White arrowheads show typical dorsomarginal kineties. (B) Posterior region. Asterisks mark spaced dorsal bristles, possibly parental structures, at the field of scattered dikinetids. (C) Specimen with one dorsal bristle (white asterisk) between dorsal kineties 3 and 4. Black asterisk marks kinety 4. (D) Anterior region of specimen with three typical dorsomarginal kineties (arrowheads). (E) Detail of dorsal dikinetid showing that anterior basal body is ciliated. (F) Detail of caudal cirri to show barren basal bodies (arrowheads). Scale bars: A, B, D. 10 μm; F. 3 μm.

Adoral zone of membranelles occupying about 42% of total body length (calculated on the average values measured on protargol-impregnated cells). Buccal cavity rather large and deep, with a conspicuous buccal lip covering proximal membranelles. Adoral zone with 50–62 membranelles, with lateral membranellar cilia extending to the right of the buccal cavity; distal membranelles about 13 μm long. Paroral and endoral intersect each other optically behind buccal cirrus, arranged in the *Cyrtohymena*-pattern; both membranes composed of dikinetids. Somatic ventral ciliature composed of two ventral rows of ~ 11 μm long cirri, three strong, ~ 14 μm long frontal, two frontoventral and one ~ 11μm long buccal, usually one ~ 12 μm long postperistomal, two ~ 13 μm long pretransverse, and 5–8 ~ 15 μm long transverse cirri. One right and one left marginal row of cirri. Right ventral row begins near distal end of adoral zone of membranelles, while left row begins behind frontoventral cirri. Right marginal row begins almost at the level of right frontal cirrus and terminates at the level of posteriormost transverse cirrus; left marginal row terminates conspicuously behind the level of right marginal row. Transverse cirri not protruding conspicuously beyond posterior end of cell. Marginal and ventral cirri with leftmost basal bodies barren; some barren basal bodies also in pretransverse and transverse cirri. Two or three dorsomarginal rows; leftmost dorsomarginal terminating at posterior third of body, middle dorsomarginal extends almost up to midbody, rightmost dorsomarginal formed of about two or three dikinetids, restricted to anterior region of body. Dorsal bristles about 2.5 μm long. Dorsal kineties 1 to 3 bipolar; kinety 4 short, commencing at about midbody; scattered dikinetids between kinety 3 and 4 and between kinety 3 and dorsomarginal kineties. Occurrence of scattered dikinetids varies from absent (in starving specimens) to many, when dorsal kinety 4 becomes indistinguishable. About 7–11 caudal cirri arranged in rows at rear ends of dorsal kineties 1, 2, and 4. Usually 2–4 caudal cirri at end of kinety 1, 2–3 at end of kinety 2, and 3–7 at end of kinety 3 (Figs 3A–3C, 4A and 4B, 5A–5D, 6A–6C and 7A–7F). Anteriormost caudal cirri with at least one barren kinetosome (Fig 7F).

### Notes on behavior and autoecology

**Fig 8:**
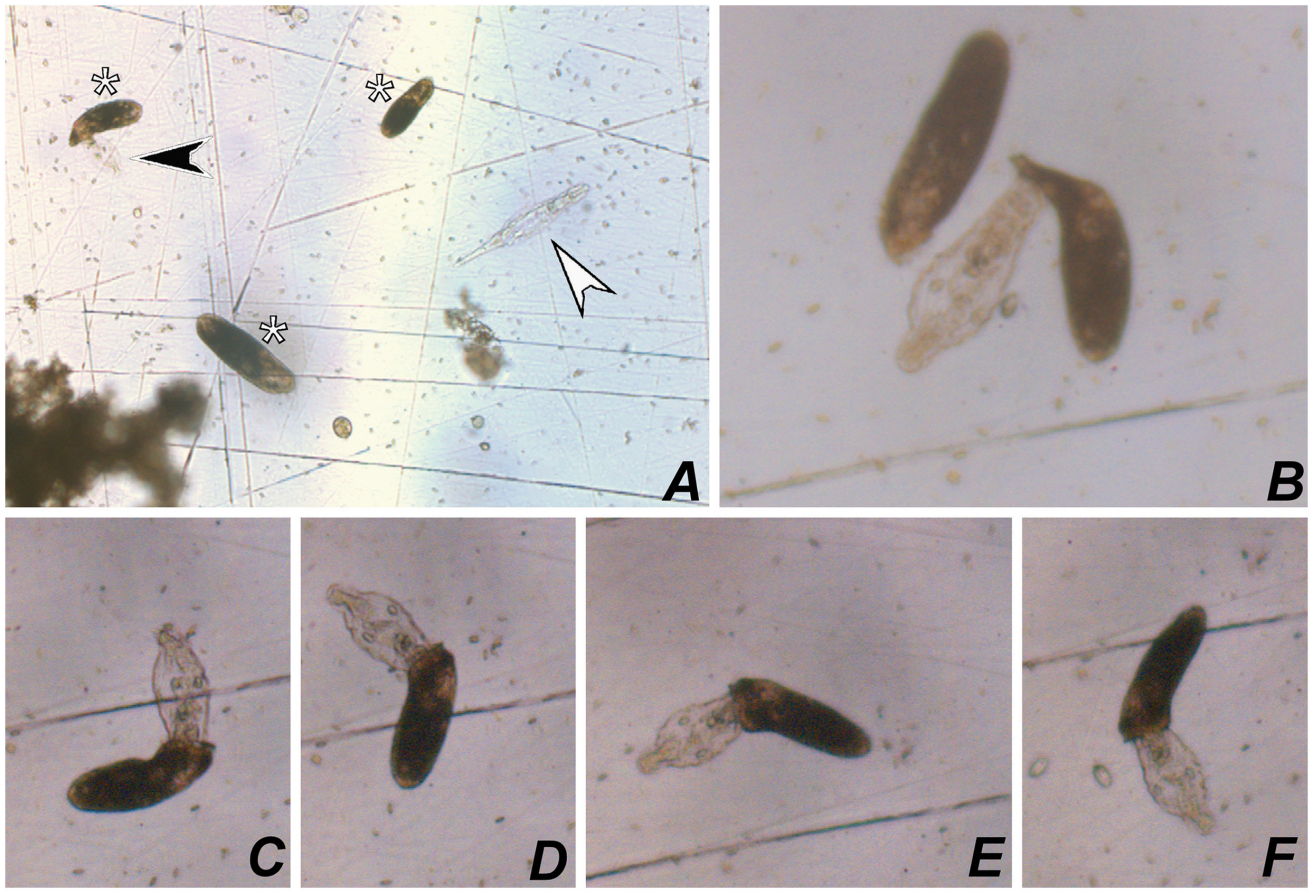
Photomicrograhps of *Apoamphisiella vernalis* from life, under stereomicroscope. (A) Specimens of *A*. *vernalis* (asterisks). Black arrowhead marks a dead rotifer being sucked by a specimen of *A*. *vernalis*; white arrowhead shows a live rotifer. (B–F) Specimen of *A*. *vernalis* attempting to swallow a dead rotifer.

Specimens move moderately fast, crawling on the bottom of Petri dishes, sometimes swimming in the water column. When the Petri dish is manually agitated, specimens become re-suspended and tend to immediately swim back to the bottom. *Apoamphisiella vernalis* is omnivorous, feeding on bacteria and protists, such as *Vorticella* sp. We frequently noticed *A*. *vernalis* attempting to feed on moribund or dead bdelloid rotifers of comparable size. Specimens of *A*. *vernalis* grabbed rotifers with the oral cavity, however were not able to swallow the whole animal. Instead, they moved around while carrying it, releasing it afterwards and repeating the process, slowly breaking and consuming parts of the animal (Fig 8A–8F). As observed by Paiva *et al*. [39] specimens of *A*. *vernalis* can also ingest arcelline testate amoebae, flagellates and ploimid rotifers. Average water characteristics in thriving cultures from the population of PA were: pH = 7.8; O_2v_saturation = 69.3%; salinity = 0.1 ppt; ORP = 0.41 mV; temperature = 26°C.

### Divisional morphogenesis

**Fig 9:**
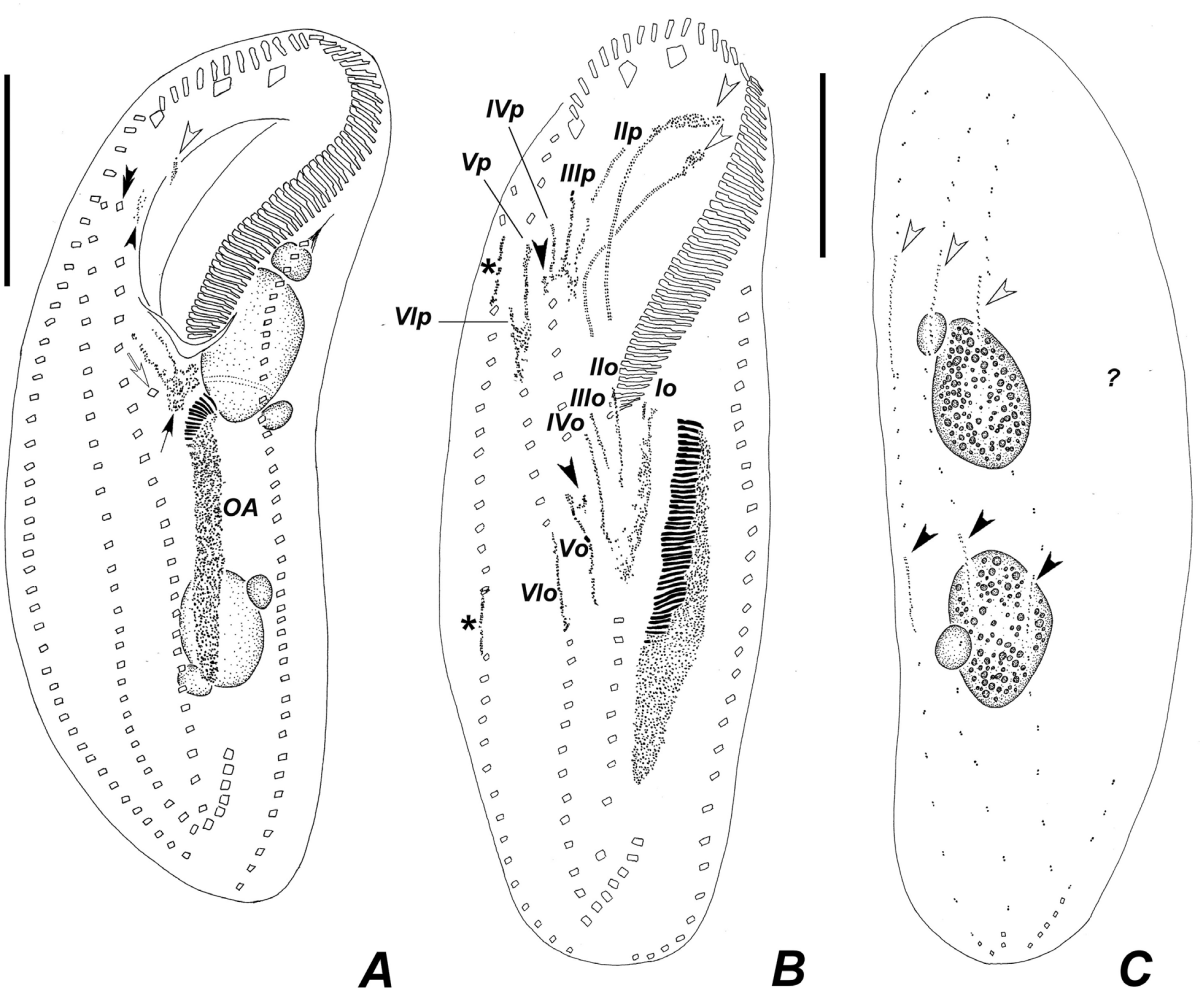
Diagrammatic representations of protargol-impregnated specimens of *Apoamphisiella vernalis* showing stages of divisional morphogenesis. (A) Ventral side of an early divider showing oral anlage and early development of fronto-ventral-transverse anlagen. White arrowhead shows disaggregating buccal cirrus; black arrowhead shows disaggregating left frontoventral cirrus; double arrowhead points to right frontoventral cirrus, still intact at this stage. White arrow shows postperistomal cirrus; black arrow shows early fronto-ventral-transverse anlagen of the opisthe. (B) Ventral side of a middle divider showing fronto-ventral-transverse anlagen (numbered) and right marginal anlagen (asterisks). White arrowheads shows disaggregating endoral and paroral; black arrowheads show extra anlagen originating between anlagen IV and V of both proter and opisthe. (C) Dorsal side of the same divider showing dorsal kineties anlagen of proter (white arrowheads) and opisthe (black arrowheads). OA = oral anlage; ? = insufficiently impregnated region. Anlagen labeled with roman numbers, where “o” names the opisthe and “p” names the proter. Scale bars: 50 μm.

**Fig 10:**
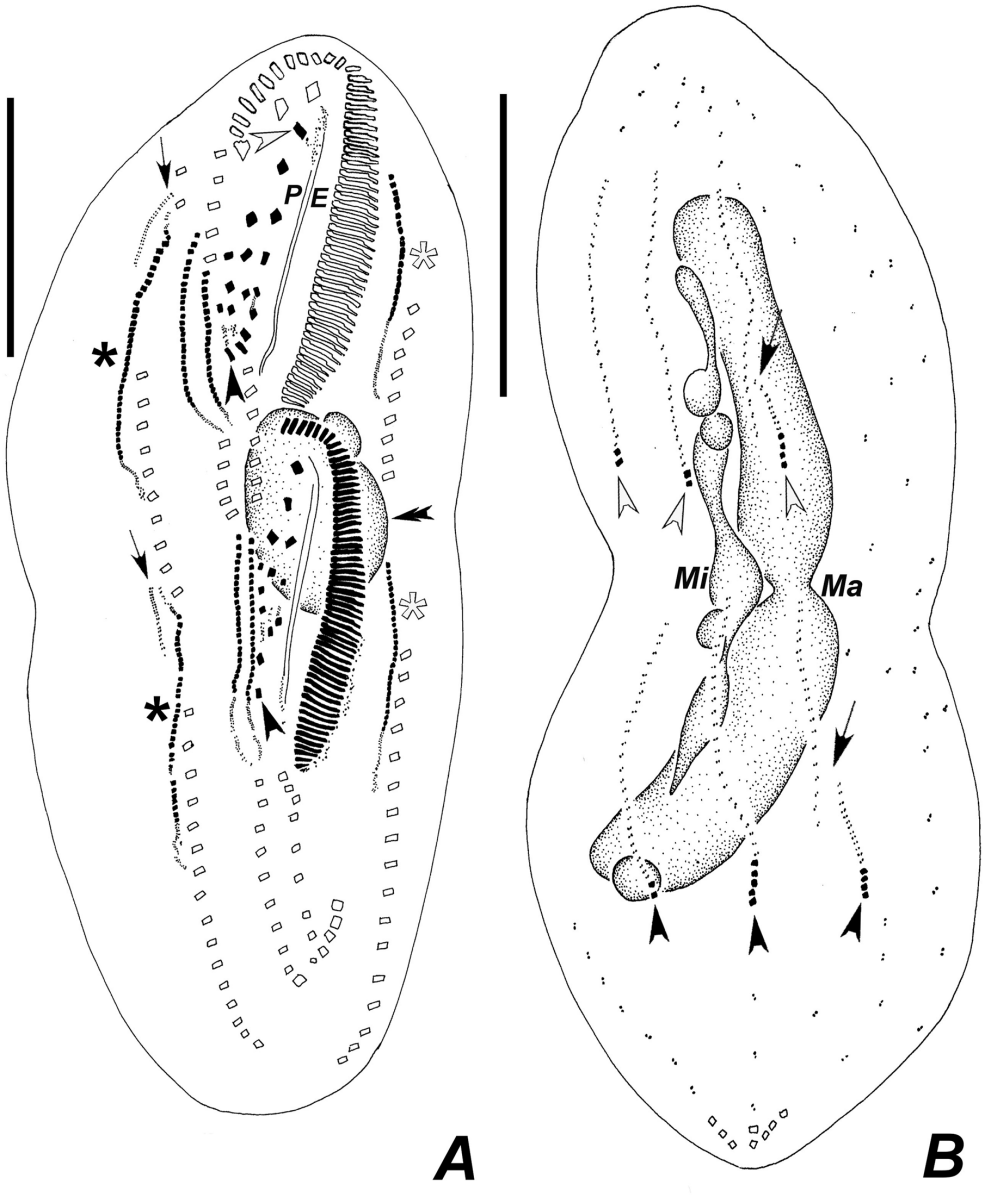
Diagrammatic representations of protargol-impregnated specimens of *Apoamphisiella vernalis* showing stages of divisional morphogenesis. (A) Ventral side of middle divider. White arrowhead shows left frontal cirrus segregating from undulating membranes anlage; black arrowheads show formation of transverse cirrus by each of the extra anlagen of proter and opisthe; white and black asterisks mark left and right marginal rows anlagen, respectively, for proter and opisthe. Dorsomarginal anlagen shown by arrows. The double arrowhead shows macronuclear nodules fused into a single mass. (B) Dorsal side of same divider, showing juvenile caudal cirri of proter (white arrowheads) and opisthe (black arrowheads), fragmentation of rightmost dorsal anlage (arrows), and dividing macronucleus and micronuclei. E = endoral; Ma = macronucleus; Mi = micronucleus; P = paroral. Scale bars: 50 μm.

**Fig 11:**
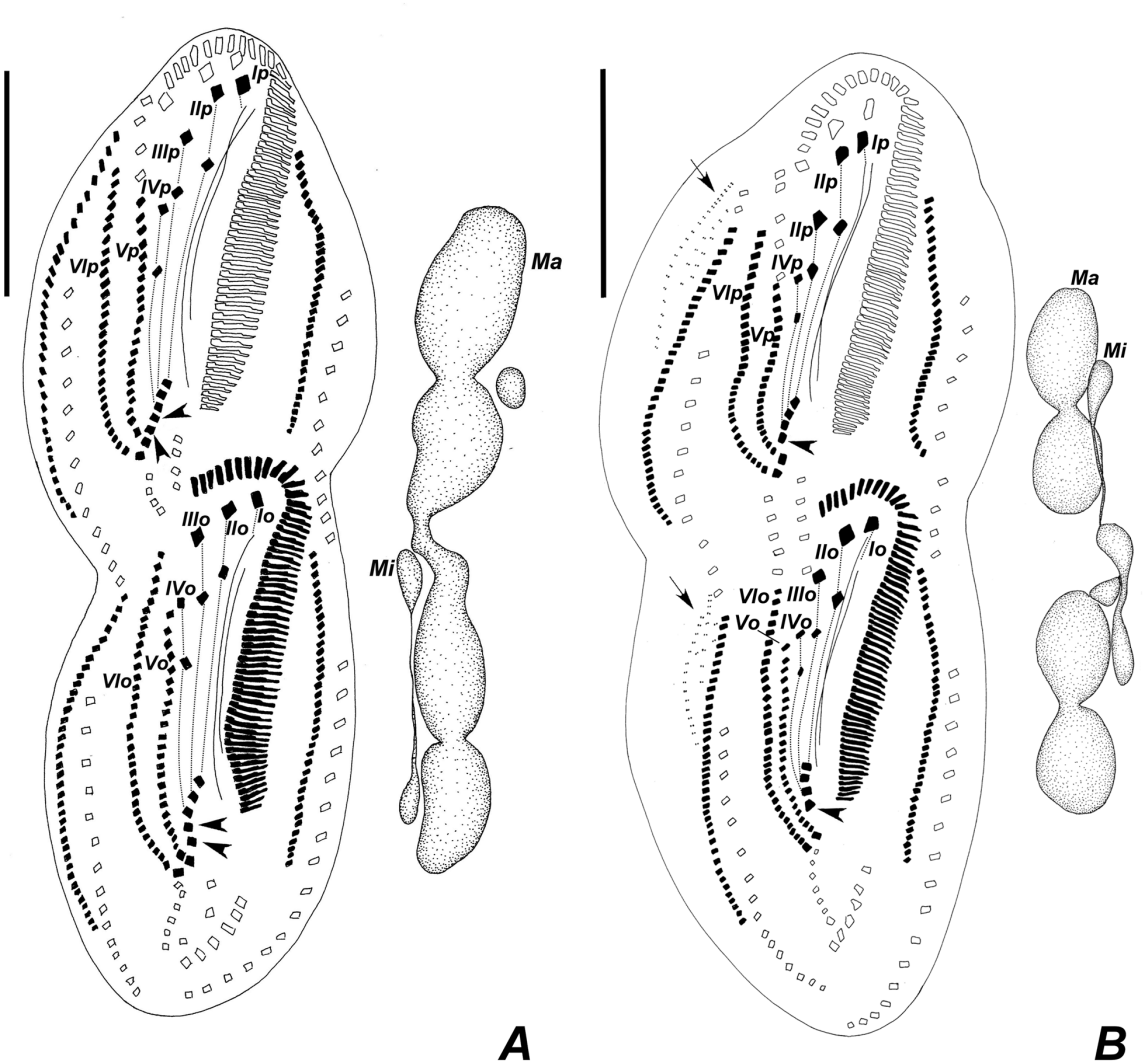
Diagrammatic representation of protargol-impregnated late dividers of *Apoamphisiella vernalis* showing dividing macronucleus and micronuclei, and ventral side with juvenile fronto-ventral-transverse cirri linked according to anlagen and numbered. (A) Specimen with two extra transverse cirri (arrowheads). (B) Specimen with one extra transverse cirri and with dorsomarginal anlagen visible (arrows). Ma = macronucleus; Mi = micronuclei. Anlagen labeled with roman numbers, where “o” names the opisthe and “p” names the proter. Scale bar: 50 μm.

**Fig 12:**
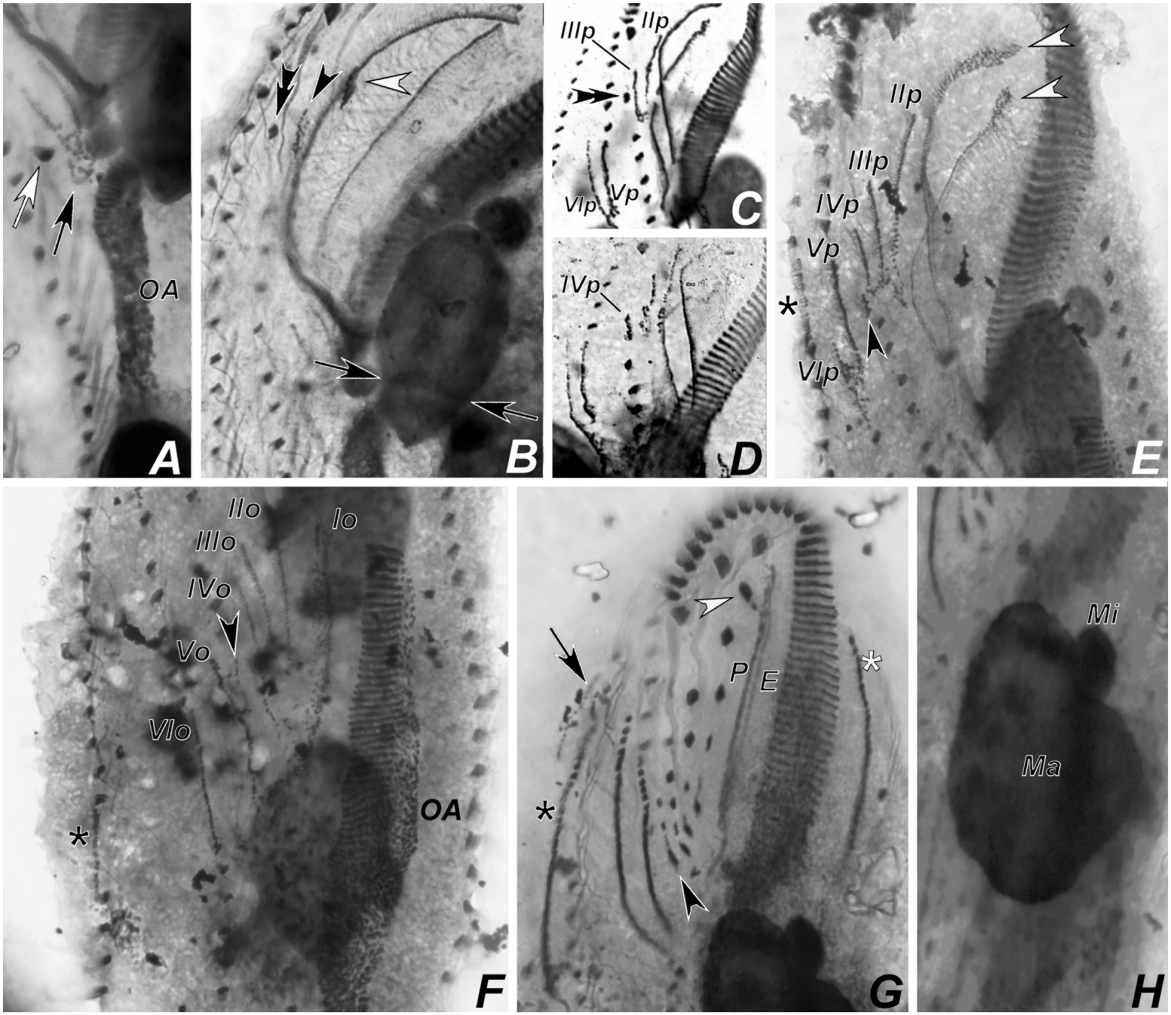
Photomicrograhps of *Apoamphisiella vernalis*, after protargol-impregnation, showing stages of divisional morphogenesis of ventral side. (A) Early divider showing oral anlage. Black arrow shows early development of fronto-ventral-transverse anlagen of opisthe; white arrow shows postperistomial cirrus. (B) Anterior region of same specimen showing disaggregating buccal (white arrowhead) and left frontoventral cirrus (black arrowhead). Double arrowhead shows right frontoventral cirrus. Arrows show macronuclear DNA replication band. (C) and (D) Specimens from Minas Gerais showing origin of anlage IV of the proter from disaggregation of right frontoventral cirrus (double arrowhead). (E) Anterior region of middle divider showing proter’s anlagen. White arrowheads show disaggregating undulating membranes. (F) Detail of morphogenesis of the opisthe in the same divider. Black arrowheads and asterisks in C and D show extra anlagen originating between anlagen IV and V, and right marginal row anlage, respectively, for proter and opisthe. (G) Proter of middle divider. White arrowhead shows juvenile left frontal cirrus detaching from undulating membranes anlage; black arrowhead points to juvenile transverse cirrus developing from extra anlage formed between anlagen IV and V. White and black asterisks mark left and right marginal anlagen, respectively; arrow marks dorsomarginal kinety anlage. (H) Same specimen, focal plane of nuclear apparatus, to show macronucleus as a single mass and dividing micronucleus. E = endoral; Ma = macronucleus; Mi = micronucleus; OA = oral anlage; P = paroral. Anlagen labeled with roman numbers, where “o” names the opisthe and “p” names the proter.

**Fig 13:**
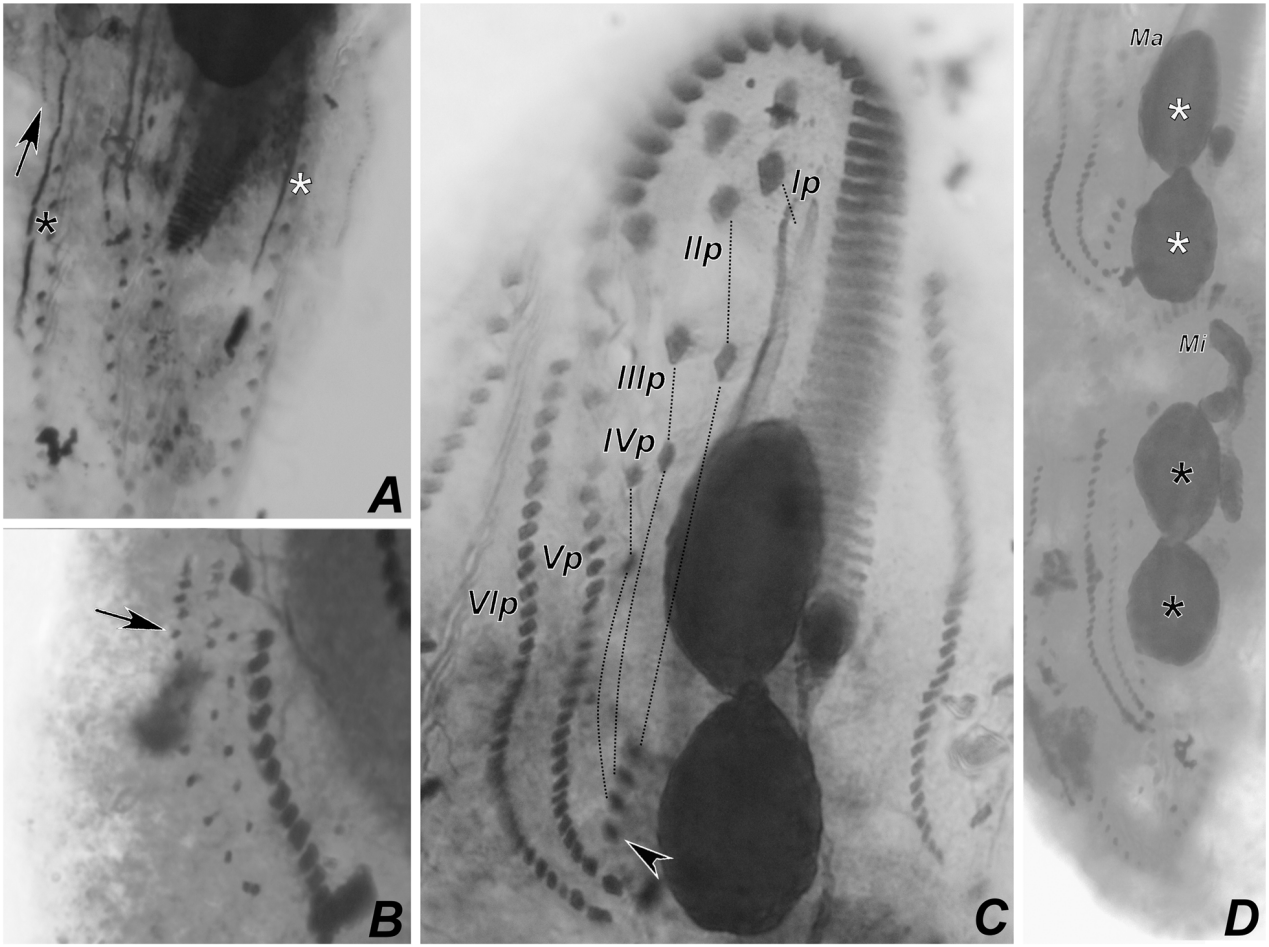
Photomicrograhps of *Apoamphisiella vernalis* after protargol-impregnation, showing stages of divisional morphogenesis of ventral side. (A) Opisthe of middle divider showing left (white asterisk) and right (black asterisk) marginal rows anlagen and dorsomarginal kineties anlagen (arrow). (B) Detail of juvenile dorsomarginal kineties (arrow) of opisthe. (C) Proter of late divider showing juvenile fronto-ventral-transverse cirri linked according to anlagen. Black arrowhead shows transverse cirrus formed by extra anlage. (D) Late divider showing distribution of juvenile macronuclear nodules in proter (white asterisks) and opisthe (black asterisks). Ma = macronuclear nodule; Mi = micronucleus.

**Fig 14:**
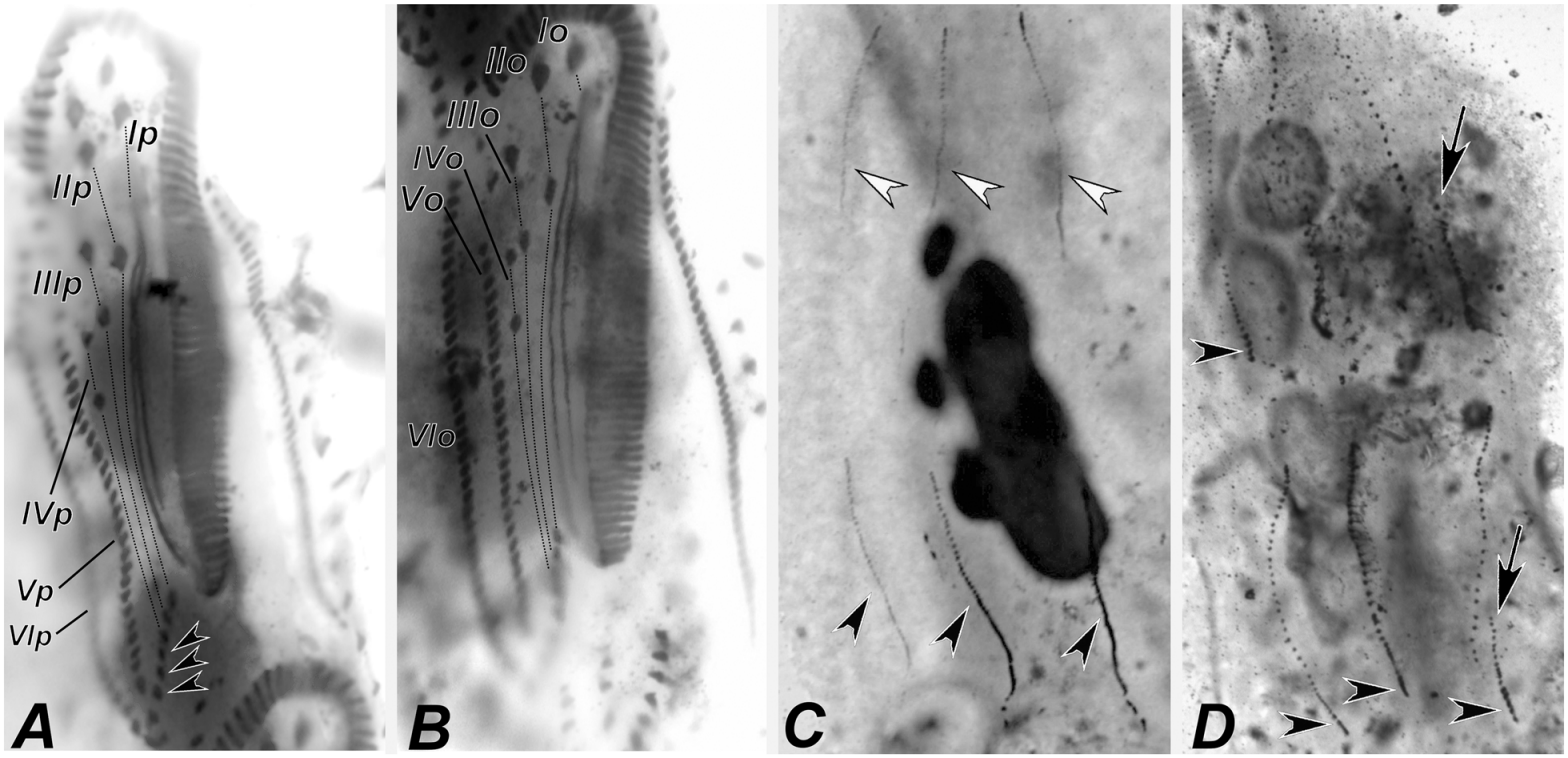
Photomicrographs of *Apoamphisiella vernalis* from Minad Gerais after protargol-impregnation, showing stages of divisional morphogenesis. (A) and (B) Ventral side of middle-to-late divider showing anlagen for proter (A) and opisthe (B). Arrowheads mark surplus transverse cirri in the proter (notice that surplus transverse cirri are also present in the opisthe, but out of focus in the image). (C) Middle divider showing dorsal kineties anlagen for the proter (white arrowheads) and opisthe (black arrowheads). (D) Fragmentation of right dorsal anlagen (arrows) and formation of caudal cirri (arrowheads). Anlagen labeled with roman numbers, where “o” names the opisthe and “p” names the proter.

**Fig 15:**
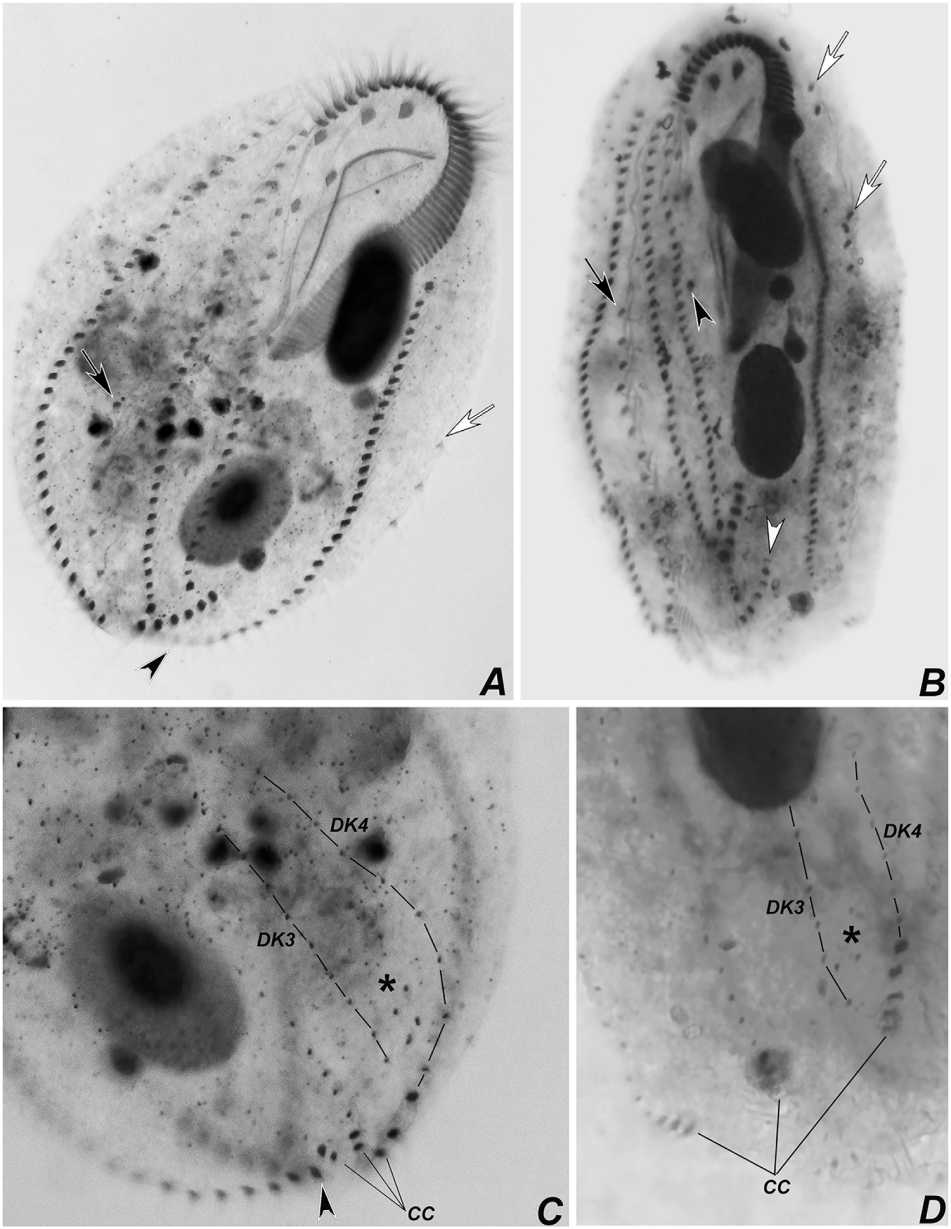
Photomicrograhps of postdividers of *Apoamphisiella vernalis*, after protargol-impregnation. (A) Ventral side of proter postdivider. Black arrowhead points to terminus of left marginal row. White and black arrows show parental left and right marginal cirri, respectively. (B) Ventral side of opisthe postdivider. Black arrowhead shows postperistomal cirrus, which did not yet reach its final position behind buccal vertex. White arrowhead shows parental transverse cirri. White and black arrows show parental left and right marginal cirri, respectively. (C) Dorsal side of specimen from A, with black arrowhead showing terminus of left marginal row. (D) Dorsal side of specimen from B. Asterisks in C and D mark scattered dikinetids between dorsal kineties 3 and 4, possibly resulting of late multiple fragmentation of rightmost dorsal anlage. CC = caudal cirri; DK(n) = dorsal kinety.

Stages of divisional morphogenesis in typical specimens (Figs 9A–9C, 10A, 10B, 11A, 11B, 12A–12H, 13A–13D, 14A–14D and 15A–15D) of both populations were identical. Stomatogenesis begins likely *de novo*, in the area between the left ventral and left marginal row. Enlargement by basal bodies proliferation result in an elongated anarchic field of basal bodies. Adoral membranelles differentiate backwards and FVT anlagen II and III of the opisthe develop from a patch of basal bodies near the anterior end of the oral anlage, as two streaks that proliferate anteriad. Postperistomal cirrus probably dedifferentiates and forms anlage IV of the opisthe. At this time, buccal and left frontoventral cirri of the proter disaggregate to generate FVT anlagen II and III, respectively; anterior macronuclear nodule shows a replication band (Figs 9A, 12A and 12B).

Presumptive undulating membranes of the opisthe align longitudinally and parallel to the forming adoral zone; anlagen I, II, III and IV elongate right of the oral primordium. Cirri from right and left ventral rows then disaggregate to generate FVT anlagen VI and V of the opisthe, respectively. Later, those become divider’s ventral rows. Differentiation of adoral membranelles continues backwards. In the proter, parental undulating membranes dedifferentiate and FVT anlagen II and III lengthen. Anlage IV arises from the dedifferentiation of right frontoventral cirrus, which is shown in Fig 12C–12E. Both anlagen V and VI of the proter develop from dedifferentiation of cirri from parental right ventral row. Two anlagen, one for the proter and another for the opisthe, arise within right marginal parental row (Figs 9B, 12E and 12F). The left marginal row begins to replicate slightly later than the right row, but in the same manner. Dorsal kineties anlagen develop by intrakinetal proliferation of basal bodies within kineties 1, 2 and 3 for proter and opisthe, at about the same levels of marginal rows anlagen (Figs 9B, 9C and 12C).

Later, the adoral zone of membranelles of the opisthe is almost completely formed and curved anteriorly, although some isolated basal bodies remain close to posterior membranelles; undulating membranes align in parallel, both in the opisthe and the proter. Cirral anlagen enlarge and cirri segregate from the streaks; two or three dorsomarginal anlagen develop from the right marginal row anlage. Left marginal row anlagen are also formed at this stage and macronuclear nodules fused in a single mass (Figs 10A, 12G, 12H, 13A and 13B). Next, undulating membranes curve and cirri are completely segregated; each anlage forms the following cirri: leftmost frontal cirrus segregates from the undulating membranes anlagen; anlage II forms middle frontal, buccal, and one transverse cirrus; anlage III forms right frontal, left frontoventral, and one transverse cirrus; anlage IV forms right frontoventral, postperistomal, and one transverse cirrus; anlagen V and VI develop into frontoventral rows of cirri, one pretransverse and one transverse cirrus at posterior end of each row (Figs 11A, 11B and 13C). The presence of more than six transverse cirri results from extra anlagen occurring between FVT anlagen IV and V (Figs 9B, 10A, 11A, 11B, 12C–12E, 13C, 14A and 14B). Such extra anlagen begin to segregate presumptive cirri, which except for the transverse, are reabsorbed before maturation. Dorsal kinety anlagen enlarge by intrakinetal proliferation; rightmost dorsal anlage fragments and forms both kineties 3 and 4. Several caudal cirri are formed at termini of kineties 1, 2, and 4 (Figs 10B, 14C and 14D). Macronucleus elongates and constricts to form two masses that later constrict again and form two nodules for each divider; micronuclei divide by mitosis (Figs 10B, 11A, 11B and 13D). Parental adoral zone of membranelles remains intact and frontal, pretransverse, and transverse cirri do not participate in anlagen formation. In postdividers, postperistomal cirrus has not yet reached its final position behind the buccal vertex and dorsally scattered dikinetids can be seen right of kinety 3. Postdividers still retain some parental structures (Fig 15A and 15B), most remarkably, those from the opisthe, have old pretransverse, transverse and marginal cirri, which are later fully reabsorbed (Fig 15B). Possibly, late multiple fragmentation of rightmost dorsal anlage occurs prior cytokinesis in some specimens, resulting in a few scattered dikinetids between dorsal kineties 3 and 4. The scattered field of dikinetids between kinety 4 and dorsomarginal kineties is likely formed by retained parental structures (Fig 15C and 15D).

**Fig 16:**
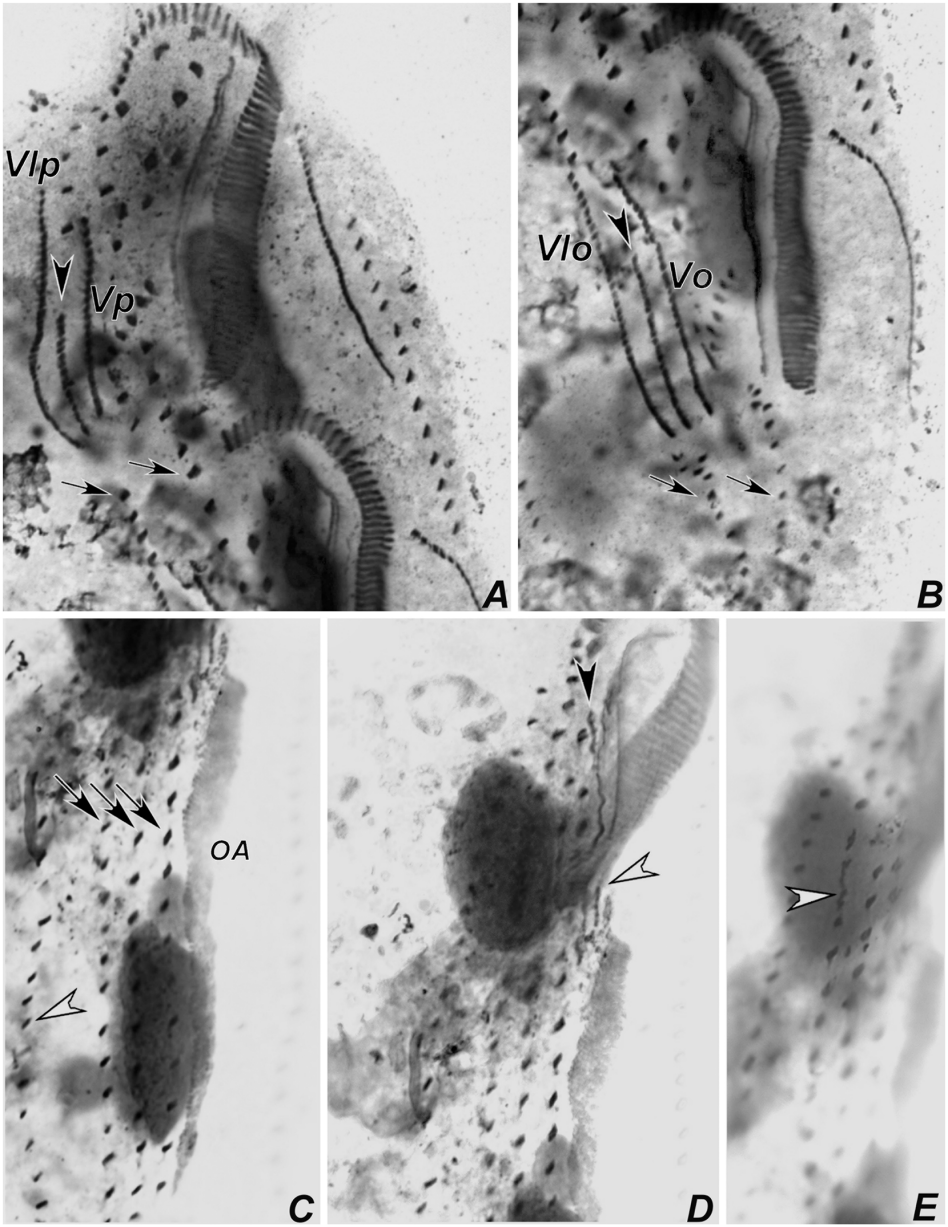
Photomicrograhps of *Apoamphisiella vernalis* from Minas Gerais, after protargol-impregnation, to show variations in morphogenesis pattern of ventral ciliature. (A) Proter and (B) opisthe of middle divider with two ventral cirral rows (arrows) producing one extra anlage between each anlagen V and VI (arrowheads); arrows point to parental ventral rows. (C–E) Specimen with three ventral cirral rows (arrows) at different focal planes. Arrowhead in C points to right marginal cirral row; arrowhead in D shows early development of undulating membranes anlage of opisthe; arrowhead in E points to anlage formation within rightmost ventral row of opisthe. OA = oral anlage; anlagen labeled with roman numbers, where “o” names the opisthe and “p” names the proter.

**Fig 17:**
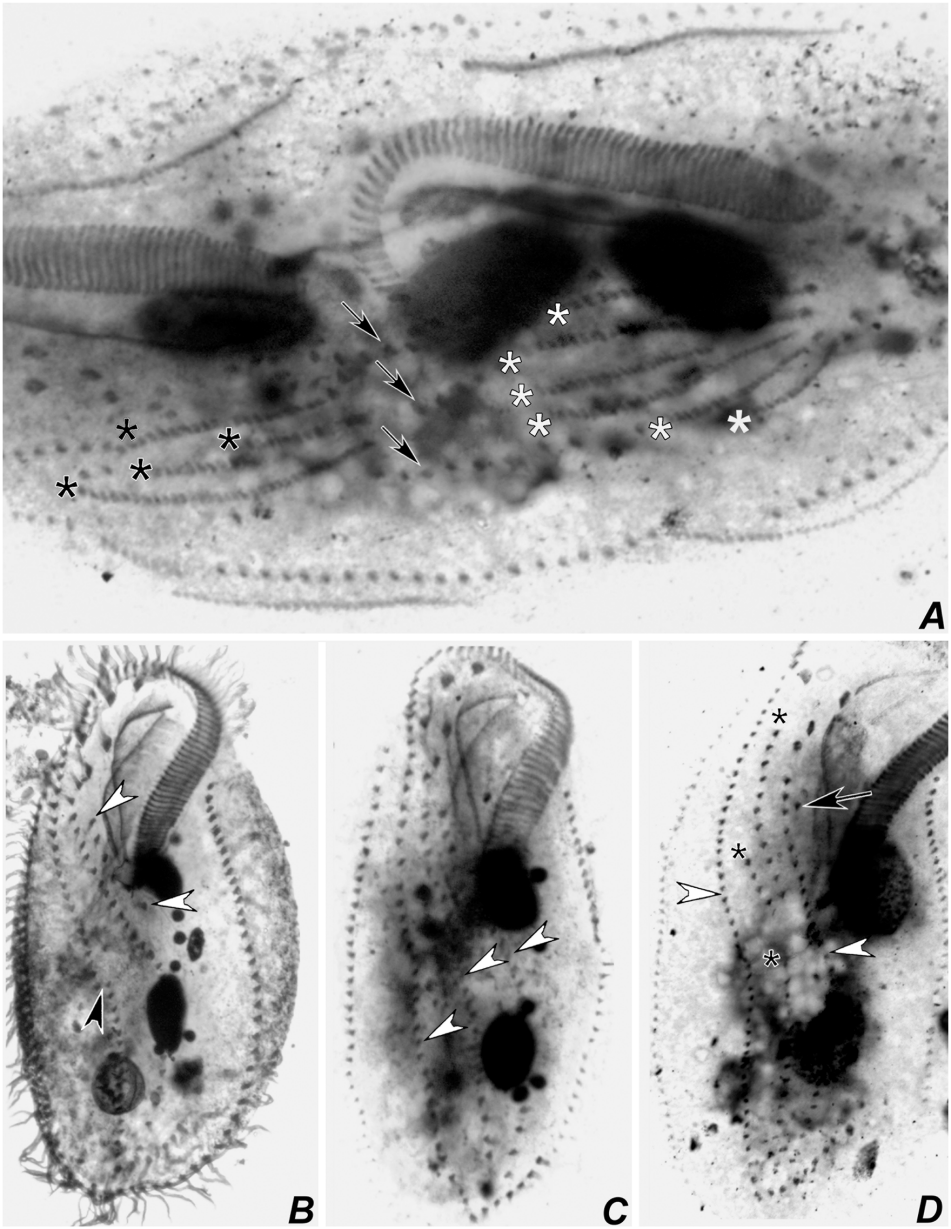
Photomicrograhps of *Apoamphisiella vernalis* from Minas Gerais, after protargol-impregnation, to show variations in morphogenesis pattern of ventral ciliature. (A) Middle divider (oriented with anterior end of cell towards left side of the page) with three ventral cirral rows (arrows) producing at least four juvenile ventral rows for the proter (black asterisks) and six for the opisthe (white asterisks). (B–D) Interphase specimens resulting from dividers with deviant morphogenetic patterns. (B) Specimen with a truncated (amphisiellid?) right ventral row (black arrowhead) and increased number of cirri produced by anlage IV, forming a row homologous to postperistomal cirrus (white arrowheads). (C) Specimen with at least three long ventral rows (arrowheads). (D) Specimen with shortened ventral rows (asterisks) between long ventral rows supposedly homologous to left and right ventral rows of typical specimens (white arrowheads). Black arrow shows extra cirrus behind right frontoventral cirrus.

In the population from MG, 36 out of 80 specimens were atypical, because they had more than two long ventral cirral rows, which is explained by deviations in the morphogenetic processes. Occasionally, specimens with typical morphology produce an extra anlage that grows between anlagen V and VI (Fig 16A and 16B). This extra anlage forms a middle ventral cirral row. Next, specimens with three ventral rows develop multiple ventral anlagen, each forming a slightly short or fragmentary ventral row (Figs 16C–16E and 17A). In addition, in specimens with more than two ventral cirral rows, the number of cirri produced by anlage IV seems to be increased, thus forming a row immediately right of the peristome, which ends at the postperistomal region. One interphasic specimen (out of 80) had a truncated right ventral row, suggesting that sometimes, this may be an amphisiellid structure formed by two anlagen (Fig 17B–17D).

### Phylogenetic analyses

The sequence of *A*. *vernalis* from PA (KU522216) was 1,770 nucleotides long, with GC content of 42.8; the sequences from MG, KU522214 and KU522215, were 1,765 and 1,769 nucleotides long, and had GC content of 42.5 and 42.6, respectively. The *P* distance between the sequences from MG was of 0.002, and of 0.004 between those from MG and PA (Table 2). The complete nucleotide matrix had 1,883 characters (of which 44.5% were identical) after aligning and trimming.

**Fig 18:**
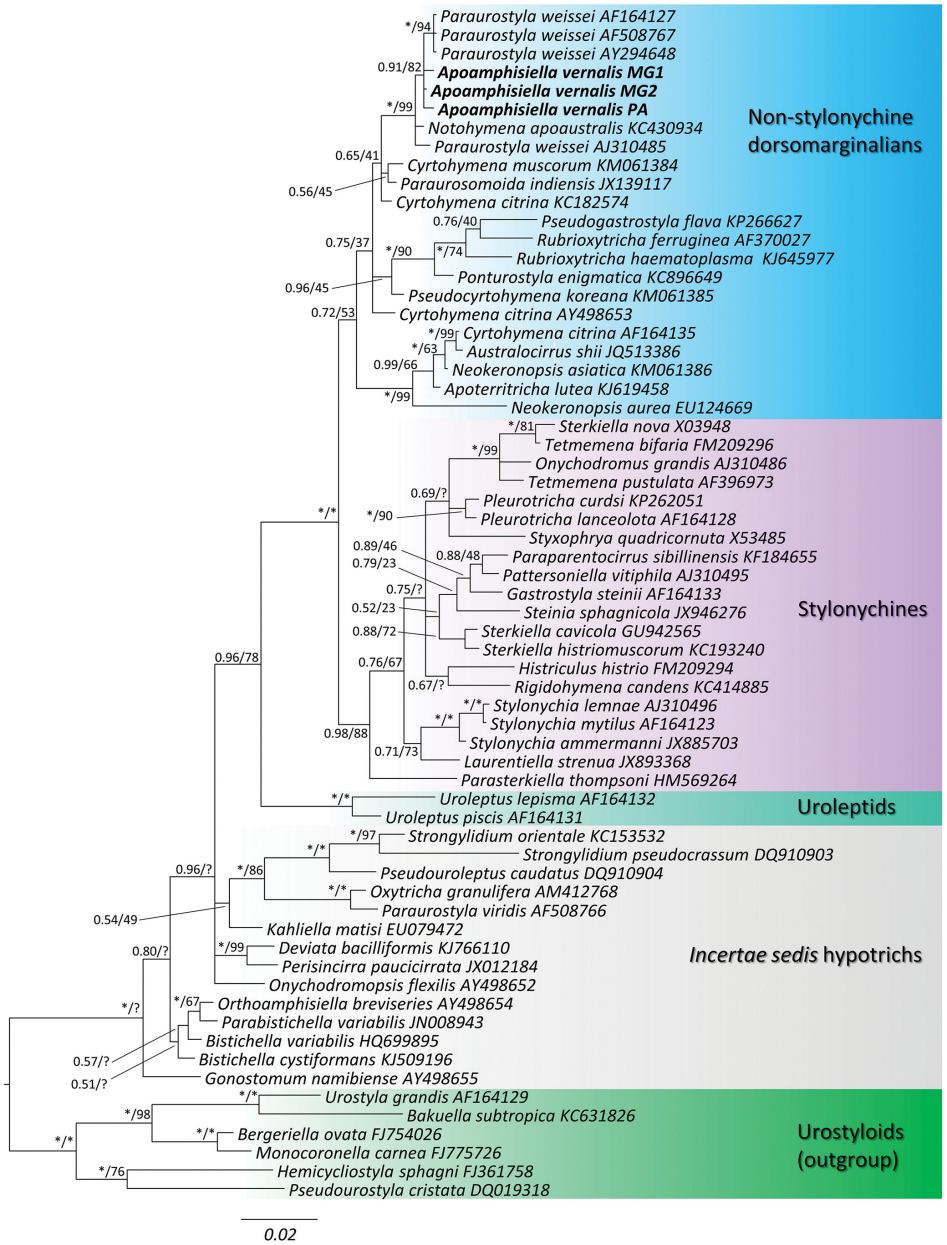
Bayesian inference (50% majority rule consensus) tree of Hypotricha, emphasizing the relationships within the Dorsomarginalia (uroleptids, stylonychines and “non-stylonychine dorsomarginalians”) and some *incertae sedis* representatives. Values associated to nodes are Bayesian posterior probabilities / maximum-likelihood bootstrap support. * = full support; ? = node contradicted in the maximum-likelihood tree. Scale bar = substitutions per 100 nucleotide positions.

The topology of the ML tree differed slightly from that of the BI (Fig 18) in some weakly (Bayesian posterior probability < 0.90; bootstrap < 50%) supported clusters. Namely, in the ML tree, *Gonostomum namibiense* diverged after *Bistichella* spp., *Parabistichella variabilis* and *Orthoamphisiella breviseries*, which formed a pectinated pattern; *Histriculus histrio* branched off the base of the *Tetmemena* spp. + *Onychodromus grandis* + *Sterkiella nova* cluster; and *Notohymena apoaustralis* was sister terminal of *P*. *weissei* from Austria (AJ310485) (bootstrap = 48%). In both BI and ML analyses, the selected ingroup was monophyletic in relation to the selected outgroup. The affinities among the *incertae sedis* hypotrich terminals had weak data support and were poorly resolved, except for the placement of *Oxytricha granulifera* + *Paraurostyla viridis* as an adelphotaxon of the *Pseudouroleptus* + *Strongylidium* cluster; and the monophyly of the cluster formed by *Deviata bacilliformis* and *Perisincirra paucicirrata*. The Dorsomarginalia were polyphyletic due to the position of the oxytrichids *Oxytricha granulifera* and *Onychodromopsis flexilis* outside the main dorsomarginalian cluster, which was formed by the uroleptids plus the stylonychines and remaining flexible-body oxytrichids, herein called “non-stylonychine dorsomarginalians” (NSD).

The *Apoamphisiella vernalis* terminals were unambiguously placed within the NSD in a polytomic, but strongly supported, cluster with the strains of *Paraurostyla weissei* from USA. This cluster was related to *P*. *weissei* from Austria, *N*. *apoaustralis*, *Paraurosoma indiensis*, *Cyrtohymena muscorum*, and the Indian strain of *Cyrtohymena citrina*.

The statistical tests for comparisons among competing phylogenetic trees (Table 3) rejected the null hypothesis for the constrained topology scenarios I and III; hence, trees enforcing a monophyletic resolution for the *Paraurostyla weissei* complex, either alone or with *A*. *vernalis*, were not significantly different from the ML tree. On the other hand, trees enforcing the monophyly of all sequences labeled as *Paraurostyla* were significantly different from the ML tree.

**Table 3:** *P* values of statistical tests for tree comparisons considering different topological constraint scenarios.

	Constrained topologies	AU	SH	WSH
***I***	*Paraurostyla weissei* complex + ***Paraurostyla viridis***	1e-094	0.000	0.000
***II***	*Paraurostyla weissei* complex	0.063	0.676	0.419
***III***	*Apoamphisiella vernalis + Parauostyla weissei* complex + ***Paraurostyla viridis***	4e-058	0.000	0.000
***IV***	*Apoamphisiella vernalis* + *Paraurostyla weissei* complex	0.565	0.917	0.917

AU = approximately unbiased; SH = Shimodaira-Hasegawa; WSH = weighted Shimodaira-Hasegawa. α = 0.01

## Discussion

### Comparison with related species

Currently, the genus *Apoamphisiella* comprises five species. Those are *A*. *tihanyiensis* (Gellért & Tamás, 1958) Foissner, 1997 (type species), *A*. *hymenophora* (Stokes, 1886) Berger, 1999; *A*. *jurubatiba* Paiva & da Silva-Neto, 2003, *A*. *foissneri* Paiva & da Silva-Neto 2004; and *A*. *vernalis*. As pointed by Paiva *et al*. [39], common taxonomic features related to body size and ciliary structures overlap among species of *Apoamphisiella* [67–71], the most stable characters being presence/absence and color of cortical granules, number of contractile vacuoles and presence/absence of collecting canals. Within this context, the presented population was tentatively identified as *A*. *vernalis* sensu Paiva *et al*. [39] at morphospecies level because of the presence of green-colored cortical granules and a single contractile vacuole without noticeable collecting canals. Moreover, the specimens from PA have marginal and ventral cirri with the leftmost basal bodies barren, as was also observed in the population from MG [39]. It is worthy of note that although the barren basal bodies could result from preparation artifact, we find this unlikely, because they appeared consistently in the preparations, and did not occur in other hypotrichs fixed according to the same protocol adopted herein.

Some characteristics must be brought into attention because they differ from those observed in the population from MG, described in detail by Paiva *et al*. [39]. We found the cytoplasm of *A*. *vernalis* from PA to be much less “rugged” in appearance than reported by Paiva *et al*. [39]. The ruggedness observed in the population from MG is due to the presence of many polygonal crystals densely packed inside of almost the whole cytoplasm, rendering the body an opaque appearance [39]. Such polygonal crystals are scarcely present in specimens from PA (see Fig 2E), which in addition, contained conspicuous aculeate refractive crystals that were absent in the population from MG studied by Paiva *et al*. [39]. We also noticed that specimens from PA glide quickly on glass Petri dishes, contrarily to the slow moving specimens from MG population [39]. As an additional remark, in the population from PA, the LMR ends below the level of RMR (*vs*. at same level in the MG population). An exam of protargol-impregnated slides of other specimens from MG collected in 2006, revealed that this is a variable feature, with some specimens (often the larger ones) exhibiting the termini of LMR behind the level of RMR, as herein described. Thus; we suppose this feature varies according to nourishment conditions or even life stage, since postdividers originating from the proter have the termini of both marginal rows at same level in the population from PA.

When *A*. *vernalis* from PA is compared to *A*. *tihanyiensis*, it differs mainly in the color of cortical granules (green *vs*. citrine yellow or pale yellow) and morphology of contractile vacuole (collecting canals absent or extremely inconspicuous *vs*. present and conspicuous). Foissner [67] mentioned citrine yellow cortical granules comparable to those of “*Holosticha multistilata”*, in specimens of *A*. *tihanyiensis* from soil samples of the Amazonian forest near Iquitos, Peru. He also commented on a freshwater Brazilian population from Praia do Forte (Rio de Janeiro), which had less distinctly yellowish cortical granules. Moreover, another Brazilian population, from the Cabiúnas Lagoon (Rio de Janeiro), was reported by Paiva & da Silva-Neto [69] as having pale yellow cortical granules. Just recently, Hu & Kusuoka [71] have discovered a population of *A*. *tihanyiensis* from Lake Biwa, Japan. However, its cortical granules are yellow-greenish and the dorsal ciliature contains few scattered dikinetids; features that may resemble typical specimens of *A*. *vernalis*, thus deviating from the populations of *A*. *tihanyiensis* studied by Foissner [67] and Paiva & da Silva-Neto [69], which exhibited dense fields of scattered dikinetids. Moreover, Hu & Kusuoka [71] found specimens with two postperistomal cirri (also mentioned by Foissner [67]) and some widely spaced cirri in front of the transverse set, which to our interpretation, may correspond to the similarly placed cirri depicted by Gellért & Tamás [72] in the description of *A*. *tihanyiensis* (originally *Onychodromopsis tihanyiensis*). Unfortunately, they did not provide information on the cortical granules *in vivo*, thus a more accurate comparison is not possible.

*Apoamphisiella vernalis* from PA differs from *A*. *jurubatiba* in the color of cortical granules (green *vs*. brown); and in the absence of a middle frontoventral cirrus in most specimens (sometimes it is present in the population from MG), which according to Paiva & da Silva-Neto [68, 69], was constant in *A*. *jurubatiba*. Finally, *A*. *vernalis* from PA is readily discerned from *A*. *foissneri* and *A*. *hymenophora* because both lack cortical granules [69, 70].

The terrestrial species *Paraurostyla granulifera* Berger & Foissner, 1989 has to be compared with *A*. *vernalis* because it has three ventral cirral rows and a distinct postperistomal cirrus, thus resembling the ventral pattern found in anomalous specimens of the MG population. Nevertheless, *P*. *granulifera* differs conspicuously from *A*. *vernalis* by having closely spaced, more or less ellipsoid, colorless cortical granules (*vs*. rounded, green granules) and the undulating membranes resembling the *Australocirrus*-pattern described in Kumar & Foissner [73] (*vs*. cyrtohymenid-like). The freshwater *Trichototaxis fossicola* Kahl, 1932, which according to Berger [1] is a possible synonym of *P*. *granulifera*, has to be re-investigated before a conclusive comparison can be elaborated. Even though, the presence of three ventral rows, if proven constant, differs from the common condition found in *A*. *vernalis*, viz. two of such structures. Moreover, the dorsal ciliature of both *P*. *granulifera* and *T*. *fossicola* still remain to be described [1, 74, 75].

The phylogenetic tree presented here shows that *A*. *vernalis* is very closely related to the *Paraurostyla weissei* complex and *Notohymena apoaustralis*; thus, these species should also be compared at morphological level. Even considering the atypical specimens found in the population from MG, *A*. *vernalis* can be readily discerned from the Austrian strain of *P*. *weissei* by the presence of invariably three frontal cirri (*vs*. invariably four); and from the North American strains by the relative position of the anterior ends of the ventral cirral rows (LVR or second rightmost ventral row below or at least at the level of right frontoventral cirrus *vs*. conspicuously above the level of right frontoventral cirrus) [1, 12, 42]. Moreover, *A*. *vernalis* differs from both Austrian and North American strains of *P*. *weissei* by the presence of a field of scattered dikinetids between DK4 and dorsomarginal kineties. Lastly, *A*. v*ernalis* differs conspicuously from *Notohymena apoaustralis* because the latter has a typical 18-FVT oxytrichid ventral cirral pattern and lacks the above mentioned dorsal field of scattered dikinetids. Such differences also apply to *Cyrtohymena citrina* and *C*. *muscorum* [41, 76, 77].

### Comparative ontogeny based on divisional morphogenesis

Hitherto, divisional morphogenesis in *Apoamphisiella* is known only for the ventral ciliature of *A*. *hymenophora* studied by Grimes & L’Hernault [78] and from a brief comment by Foissner [67], who observed that both ventral rows are involved in anlagen formation in *A*. *tihanyensis*, which is also true for *A*. *vernalis*.

According to Grimes & L’Hernault [78], the earliest event of divisional morphogenesis in *A*. *hymenophora* is the resorption of cilia along the anterior margin of the transverse cirri. In *A*. *vernalis*, we found via SEM that barren basal bodies consistently occur in the anterior margin of presumptive interphase specimens (as well as near marginal, ventral and pretransverse cirri). Hence; contraryly to *A*. *hymenophora*, those are unlike to result from resorption of pre-existent cilia prior to morphogenesis in this species. Grimes & L’Hernault [78] found that the appearance of the oral anlage in *A*. *hymenophora* occurs by proliferation of basal bodies from the anterior margin of the leftmost transverse cirrus. Unfortunately, very early dividers were not found in our preparations, thus we could not compare this stage in *A*. *vernalis*.

The remaining stages described by Grimes & L’Hernault [78] occur exactly as in *A*. *vernalis* from PA and typical specimens from MG, including the formation of at least one extra anlage on the left of ventral cirral rows, which is only involved in the production of a transverse cirrus. The formation of such extra anlagen might also happen in *A*. *foissneri*, which explains its numerous transverse cirri. According to Berger [79], this curious feature occurs in the distantly related marine *Amphisiella annulata* (Kahl, 1928) Borror, 1972 and possibly also in *Amphisiella turanica* Alekperov & Asadullayeva, 1999.

The process of divisional morphogenesis in *A*. *vernalis* are remarkably similar to those described by Jerka-Dziadosz & Frankel [12] for the North American population of the *Paraurostyla weissei* complex, which according to Berger [1], corresponds to *Paraurostyla polymicronucleata* Merriman, 1937. Some differences occur, however, in the number of anlagen and cirri produced, compared to typical specimens of *A*. *vernalis*. In this regard, *A*. *vernalis* differs from the North American *P*. *weissei* in the number of cirri produced by anlage IV that migrate to postperistomal region (1–2 *vs*. 6) and in the number of ventral rows associated to pretransverse cirri, which are formed by anlage VI (the rightmost FVT one) and anlagen that are herein interpreted as possibly serially homologous to V. Typical specimens of *A*. *vernalis* have two ventral rows (i.e., one formed by anlage V, the other by anlage VI), while North American *P*. *weissei* has 3–6 (4–7 in Jerka-Dziadosz & Frankel [12] because they counted the postperistomal as a ventral row). Remarkably, the row immediately right of the postperistomal in the North American *P*. *weissei* does not seem to be associated to a pretransverse cirrus (Fig 1A in Jerka-Dziadosz & Frankel [12] p. 615), and is posteriorly shortened, not extending up to its corresponding transverse cirrus. Thus, its originating anlage may not be serially homologous to anlage V, but perhaps related to IV. If this is correct, then such anlage is possibly homologous to the short extra anlagen from *Apoamphisiella*. This and the serial homology of anlage V is likely also true for the European *P weissei*, for which morphogenesis was described in detail by Wirnsberger *et al*. [42].

The European *P*. *weissei* has a fourth frontal cirrus, formed by the basal bodies from anterior end of anlage IV, which is less strong than those formed by anlagen I, II and III, and is invariably absent in both typical and atypical specimens of *A vernalis*. Moreover, the rightmost ventral row of the European *P*. *weissei* is amphisiellid, because its anterior segment is formed by cirri originating from anlage VI, which migrate anteriad, loosely aligning itself to the anterior end of the second rightmost ventral row [1, 42].

Among dorsomarginalian species with dorsal kinety fragmentation and two long ventral rows that end in pre-transverse + transverse cirri, the divisional morphogenesis of *A*. *venalis* should be compared with that of *Parentocirrus hortualis* Voß, 1997. In *P*. *hortualis*, about three or four cirri from anlage IV migrate to the postperistomal region, and form a line of widely spaced cirri (*vs*. only one or two closely packed postperistomal cirri positioned near the level of infundibulum vertex in *A*. *vernalis*). Additionally, in *P*. *hortualis*, the right ventral row is morphogenetically inactive (*vs*. active in *A*. *vernalis*), and both anlagen V and VI for proter and opisthe originate from disaggregating cirri in the LVR (*vs*. anlagen V and VI for proter originating from the RVR; and for the opisthe, from LVR and RVR, respectively) [77, 80].

Lastly, but not less important, the deviant cirral patterns found in the population of *A*. *vernalis* from MG result from modifications in the typical pattern of divisional morphogenesis, exhibiting characteristics of the North American and European strains of the *P*. *weissei* complex. Those are (i) the increase in the number of cirri resulting from anlage IV, forming a row adjacent to the left border of the peristome, terminating at postperistomal level; (ii) the presence of extra anlagen between V and VI, and (iii) the (rare) occurrence of a truncated, hence possibly amphisiellid, right ventral row.

### Notes on the Systematics of *Apoamphisiella* and *Paraurostyla*

Proximity between genera *Apoamphisiella* and *Paraurostyla* was already suggested by Berger [1], and is corroborated by our phylogenetic analyses. This finds additional support in the above mentioned similarities in the ontogenies of species of both genera during divisional morphogenesis. In addition, the type and organization of cortical granules, the cyrtohymenid-like arrangement of the undulating membranes [30], and the presence of dorsomarginal kineties are shared features consistent with the placement of *Apoamphisiella* within the NSD, in the *Cyrtohymena-Paraurostyla* cluster [51].

**Fig 19:**
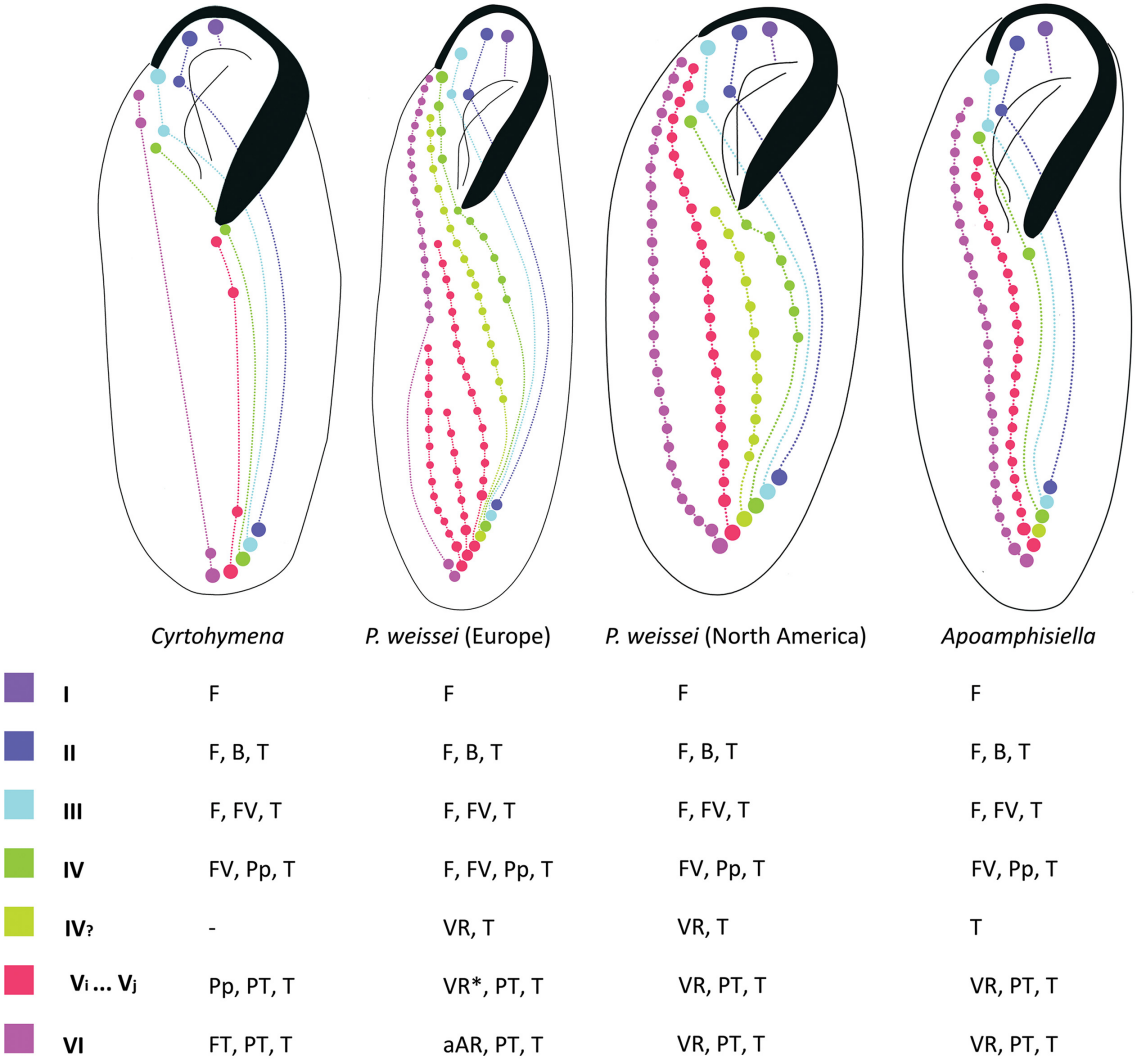
Diagram showing homologies among ventral cirri of *Cyrtohymena*, European and North American *Paraurostyla weissei*, and *Apoamphisiella* on stylized drawings. *The rightmost ventral cirral row of European *P*. *weissei* corresponds to the posterior segment of an amphisiellid row. Fronto-ventral-transverse anlagen are numbered in romans. IV_?_ = anlage supposedly related to IV; V_i_ … V_j_ = serial homology of anlage V, where “i” is the leftmost and “j” the rightmost in the series; aAR = anterior segment of the amphisiellid cirral row; B = buccal; F = frontal; FV = frontoventral; Pp = postperistomal; PT = pretransverse; T = transverse; VR = ventral cirral row; - = inapplicable.

Based on comparison of ontogenies [12, 41, 42, 76, 77], we traced homology hypotheses among the ventral cirral anlagen and their products for *Cyrtohymena* (which also applies to *Notohymena*), the North American and European *P*. *weissei* and *Apoamphisiella* (Fig 19). Within the context of the phylogenetic relationships herein hypothesized, it is feasible to propose that the patterns in *Apoamphisiella* and *P*. *weissei* evolved from a typical 18-FVT ancestor with a *Cyrtohymena*- or *Notohymena*-like body architecture. This corroborates the possibility of secondary increase in cirri number, proposed by Berger [1], p. 842, for *Paraurostyla*. Evolution from an 18-FVT archetype is thus explained by (i) the augment of cirri produced by anlage IV, V and VI; (ii) the appearance of extra anlagen right of IV; and (iii) the acquisition of repetitions of anlage V (serial homology of V). The direction of changes among the *Apoamphisiella* and the two *P*. *weissei* patterns cannot be unambiguously ascertained because of the polytomic resolution among their terminals. Worthy of notice, the field of scattered dikinetids between dorsal kinety 4 and dorsomarginal rows in the dorsal region is absent in species of *Paraurostyla* for which the dorsal ciliature is known [1, 81].

The anomalous *A*. *vernalis* from MG represented 45% of the protargol-impregnated specimens from the samples of 2006. Such anomalies were not found in previous specimens collected in 2002, studied by Paiva *et al*. [39], and neither among those from PA. Their occurrence, however cannot be overlooked, and may suggest that the stable condition of two ventral rows, a diagnostic feature of *Apoamphisiella* [67] is not fixed in the MG population of *A*. *vernalis*. The factors that may trigger the developmental modifications which result in such variations, however, are not known, and are beyond the scope of the present study.

Within the present context, this variability has systematic implications because specimens with more than two ventral rows overlap with the characterization of *Paraurostyla*, for which two key diagnostic features are: (i) the presence of three or more ventral cirral rows, and (ii) usually more than four cirri originating from the fourth to the rightmost FVT anlagen [1]. Consequently, the delimitation of both genera becomes unclear, which is supported also by the substantially short genetic distances from *A*. *vernalis* to *P*. *weissei* 18S rDNA sequences, in special to those from North America (Table 2). Remarkably, *Apoamphisiella hymenophora*, originally described as *Holosticha hymenophora* by Stokes [82], was assigned to *Paraurostyla* by Borror [25], before its transference to *Apoamphisiella* by Berger [1].

The study by Arora *et al*. [81] shows that genus *Paraurostyla* is a melting pot of heterogeneous hypotrichs that are organized in two morphotypes according to the arrangement of frontal cirri. To our view, the majority of *Paraurostyla* species and populations mentioned in the thorough review by Berger [1], which comprehends mostly the *P*. *weissei* complex, can be further (but roughly) subdivided in four subgroups. Those are the morphotypes with (i) four frontal cirri and more than three long ventral cirral rows (perhaps the rightmost row is amphisiellid), viz. the “European *P*. *weissei*” [42]; (ii) morphotypes with three frontal cirri and more than three ventral cirral rows, viz. the “North American *P*. *weissei*” [12, 83]; (iii) morphotypes with a monocorona of frontal cirri parallel to the collar of distal adoral membranelles, viz. the “African *P*. *weissei*” [84] and *P*. *coronata* Arora *et al*., 1999; and (iv) the morphotypes with three ventral rows and usually one postperistomal cirrus, viz. *P*. *granulifera* (and perhaps its supposed synonym *Trichototaxis fossicola*). Within this context, *Apoamphisiella* possibly belongs or is related to the fourth group of morphotypes.

The huge heterogeneity of *Paraurostyla* indicates it may not be a natural assemblage, as exemplified, for instance, by the highly discrepant *P*. *polynucleata*, described by Alekperov [85], which does not belong to the four morphotype subgroups mentioned above, and may be transferred to another genus in the future when new data become available [1]. In our study, the 18S rDNA sequence labeled as *P*. *viridis* in the NCBI/GenBank, clustered outside the NSD, as an adelphotaxon of *Oxytricha granulifera*, with full statistical support. Moreover, trees in which the monophyly of *Paraurostyla* was enforced (with the addition of *P*. *viridis*) were significantly different from the ML tree (Table 3). As explained in Berger [1], that population (identified as *P*. *viridis*) was not checked by a specialist in ciliate taxonomy and its position next to *O*. *granulifera* [31, 32, 63] suggests misidentification. Berger [1] commented that *P*. *viridis* sensu Kahl [74] and Pätsch [86] (originally *Urostyla viridis* Stein, 1859), resembles *Onychodromopsis flexilis*, which is related to some species of *Oxytricha* [31, 32]. *Onychodromopsis* Stokes, 1887, has extra marginal cirral rows. Misconception among long ventral rows and internal extra right marginal rows seems common mostly in the old literature, ontogeny being decisive for disambiguation.

Another important consideration is that, as has been demonstrated [29, 87–89], the systematic relevance of some morphological characters of the ventral and marginal cirral patterns may be overrated (at least for separation at genus level), and such may be the case with the two ventral cirral rows in *Apoamphisella* vs. the more than three rows in *Paraurostyla*. Given such problematic morphological and molecular circumscriptions, a systematic redefinition of *Paraurostyla* (perhaps *Apoamphisiella* should be classified as a subgenus of *Paraurostyla*) is necessary, but it must wait new ontogenetic and molecular data of other species to evaluate their genus-level taxonomy in a phylogenetic context.
